# From biological surfaces to engineered systems: how synthetic and systems biology enable biomimetic design

**DOI:** 10.3389/fsysb.2026.1860815

**Published:** 2026-09-04

**Authors:** Btisème Bendjebour, Clément Iriart, Mathumitha Jeevakumar, William Potié, Lison Salin, Sébastien Urien, Johan Habersetzer, Urielle M’Be, Sophie Mothré, Pierre-Antoine Vigneron, Julien Grimaud

**Affiliations:** 1 Graduate Program in Biotechnology Engineering, SupBiotech, Villejuif, France; 2 Bachelor’s Program in Biotechnology Engineering, SupBiotech, Villejuif, France; 3 Master’s Program in Biology and Health, Université Paris-Saclay, Orsay, France; 4 Cellule pour la Valorisation de la Recherche Étudiante, SupBiotech, Villejuif, France; 5 Institut Jean-Pierre Bourgin (IJPB), AgroParisTech, Université Paris-Saclay, INRAE, Versailles, France; 6 Laboratoire CellTechs, SupBiotech, Fontenay-aux-Roses, France

**Keywords:** adaptive materials, bioinspired design, bioinspired engineering, biological surfaces, biomimicry, synthetic biology, systems biology

## Abstract

Biological surfaces perform a wide range of functions, including communication, molecular exchange, defense, and movement. While these architectures have long inspired biomimetic surface engineering, most existing reviews focus on fabrication methods, often overlooking the biological mechanisms underlying the functions. This review examines how the structural and functional properties of biological surfaces can inform the design of advanced biomimetic systems. Using an integrative framework combining systems and synthetic biology, we establish key design principles derived from biological surfaces and illustrate their translation into engineered systems. Communication mechanisms such as directional reflection and electrochemical signaling, along with exchange processes like aquaporins or vesicle-based delivery, have informed advances in sensing, filtration, and drug delivery. Similarly, defensive strategies, including adaptive camouflage and antimicrobial surface architectures, offer opportunities for protective and responsive designs. In the context of movement, drag-reducing and adhesive surfaces have enabled innovations in robotics, smart textiles, and transport technologies. Systems biology provides quantitative, multiscale models of pattern formation, surface-mediated signaling, and organism-environment interactions, allowing the identification of key design rules. Complementarily, synthetic biology enables the engineering of living cells, tissues, and biohybrid systems capable of producing tailored surface chemistries, multiscale micro-nano architectures, and dynamic or stimuli-responsive behaviors inspired by laboratory observations. By synthesizing principles underlying surface-mediated communication, exchange, defense, and movement, this review outlines how integrating biological insight with systems-level modeling and synthetic engineering can redefine the next-generation of biomimetic designs.

## 1 Introduction

Nature has long served as a source of inspiration for technological innovation. As such, biomimicry has emerged as a powerful approach for solving complex engineering challenges. Biomimicry is the emulation of structures, processes, and systems found in nature (Benyus, 2002). One particularly rich area of biomimetic exploration lies in the study of biological surfaces, which exhibit a wide range of functionalities including water repellence, adhesion, drag reduction, and anti-fouling properties (Bhushan, 2011; Goharshenas Moghadam et al., 2021).

A biological surface is the outermost covering of a living organism or tissue with its surroundings. From the lotus leaf’s superhydrophobicity to the gecko’s dry adhesive toe pads, biological surfaces have evolved highly specialized architectures that interact efficiently with the environment (Alibardi, 2009; Choo et al., 2014). These surfaces provide valuable blueprints for designing advanced systems with improved performance across disciplines ranging from materials science and engineering to medicine and environmental technology (Ivanova et al., 2012; Verkman, 2013).

In this context, it is important to clarify the terminology used to describe engineered designs inspired by biological systems, as distinctions between these categories are not always explicit. The examples discussed in this review can be categorized according to three distinct dimensions: design strategy, composition, and functional nature. First, based on their design strategy, materials and systems may be classified as biomimetic when they aim to reproduce specific biological structures or mechanisms, preserving their structure-function relationships, whereas bioinspired designs more broadly draw on principles derived from nature without necessarily replicating the original system (Naik and Singamaneni, 2017). Based on their composition, materials and systems may be qualified as synthetic when they are made of fully abiotic components. The properties of synthetic systems are at least partly predetermined during design and fabrication as, unlike living or engineered living materials, they do not actively modify their behavior through metabolism, growth, self-repair, or adaptive biological responses after fabrication (Endy, 2005). In contrast, hybrid (or biohybrid) designs combine biological and non-biological components to achieve synergistic functionalities, sometimes integrating biomolecules or living cells within artificial matrices (Gomez-Romero et al., 2024). Finally, based on their functional nature, systems may be classified as non-living, with properties largely predetermined during design and fabrication, or living, when they incorporate metabolically active cells as functional components, enabling dynamic behaviors such as sensing, growth, and self-repair (Rodrigo-Navarro et al., 2021).

In recent years, there has been a growing body of systematic reviews focused on the biomimicry of biological surfaces, reflecting increasing interest in how biological structures inspire innovative material and surface design. However, most of this literature is primarily targeted toward audiences in engineering, materials science, or applied physics. They focus on examples of biomimetic materials (Barthlott and Koch, 2011), adhesion and friction (Gorb and Koch, 2014), or mechanical properties of biological surfaces (Gorb and Speck, 2017), all aimed either at an audience of physicists or at researchers in materials science (Goharshenas Moghadam et al., 2021; Li et al., 2024). As a result, while biological inspiration is central, the discussions often emphasize technical implementation, fabrication techniques, and performance metrics, which may limit accessibility for biologists seeking deeper insights into the underlying biological mechanisms (Ferreira et al., 2025). We therefore re-anchor engineering implementations in their originating biological constraints, namely, developmental, ecological, and mechanobiological.

Building on this biologically grounded perspective, this review positions biological surfaces within the broader frameworks of systems biology and synthetic biology. Systems biology offers complementary approaches to understand how surface properties arise from genetic, biochemical, and mechanical principles, providing models that link molecular regulation to tissue-level patterning and organism/environment interactions (Kitano, 2002). In parallel, synthetic biology enables the engineering of cells, tissues, and hybrid living materials capable of generating tailored solutions (Endy, 2005). Together, not only do these disciplines deepen our understanding of how natural surfaces are built and maintained, but they also expand the design space for biomimetic and bioinspired systems by introducing programmable, adaptive, and self-regulating functionalities. By integrating systems-level insights with synthetic design strategies, this review highlights how biological surfaces can serve both as sources of inspiration and as substrates for next-generation engineered interfaces.

Overall, this review provides a comprehensive overview of the principles of biomimicry as applied to surface design, with a focus on the structural and functional characteristics of natural surfaces. We explore the mechanisms underlying key biological surface functions and highlight current and potential advances in the fabrication of biomimetic surfaces. To this end, an examination of the biological surfaces roles in communication and exchange is first presented, considering interactions across both macro- and microscopic scales. The following part explains the defensive functions of these surfaces, focusing on their roles as decoys, physical shields, and biological or biochemical barriers. The last section reports how specific surface features facilitate movement, with particular attention to mechanisms such as superhydrophobicity, drag reduction, adhesion, and locomotion.

Beyond cataloguing biological examples and highlighting exemplar biomimetic designs, this review also highlights transferable design principles that emerge across diverse surface functions and biological systems (Box 1). These principles provide a framework for translating biological mechanisms into future biomimetic, hybrid, and engineered living designs.

Box 1 **Recurring systems and synthetic biology principles observed across the biomimetic and bioinspired examples of this review. These principles explicitly link structure, mechanism and function while making key design trade-offs clear.**

Across the case studies reviewed, effective biomimetic and bioinspired surfaces converge on a small set of transferable principles.

- Multiscale and graded architectures. Combine nano-, micro- and milli-scale features and/or gradients (roughness, modulus, porosity) to tune optics, wetting, adhesion and fracture (e.g., lotus, nacre)
- Emergent behavior. Design self-interacting devices producing organized outcomes that cannot be predicted from the properties of the individual parts alone (e.g., autonomous swarms of robots).
- Anisotropy and directionality. Orient features to drive transport or forces (drag, friction, sound/EM scattering, capillarity), enabling one-way or angle-dependent responses.
- Stimuli-responsiveness. Use triggers (humidity, pH, light, voltage, temperature) to switch states or amplify signals (e.g., chromatophores/reflections, stomatal opening).
- Trade-offs and Pareto fronts. Make design choices explicit (e.g., permeability vs. selectivity, drag reduction vs. robustness, adhesion vs. release) and report performance along these axes.
- Modularity and manufacturability. Stack layers/chemistries with distinct roles (topography, surface energy, elasticity) while favoring processes compatible with scale-up, fouling resistance and wear.
- Multifunctionality. Biological surfaces rarely perform a single task. Instead, the same architecture often simultaneously contributes to various functions (e.g., shark skin combines drag reduction and antifouling properties).

Finally, effective translation and adoption require an iterative measure-model-fabricate-validate workflow supported by multiscale imaging and *in situ* mechanical characterization to ensure standardization. In this context of biomimicry, environmental sustainability and long-term durability should also be treated as first-order design constraints.

## 2 Communication and exchange

One key function of biological surfaces is to mediate interactions across their boundaries. In this section, we distinguish two complementary processes: communication (i.e., the transmission of signals or information between organisms through biological interfaces) and exchange (i.e., the transfer of biologically relevant molecules across cells and tissues).

### 2.1 Communication

Communication is the transmission of information from one sender to one receiver (Munodawafa, 2008). While this definition may apply to various biological scales (from groups to cell compartments), we decided to focus on communication between individuals, including both pairwise and group-level interactions. Biological surfaces play various roles in the generation and conveyance of information. By integrating systems biology to model these signaling dynamics and synthetic biology to engineer programmable signaling interfaces, biomimetic surfaces can increasingly replicate the adaptive communication principles found in living organisms (Table 1).

**TABLE 1:** Biological examples and biomimetic applications discussed in this review, related to exchange and communication.

Species	Organ/body part	Mechanism/structure	Biological phenomenon	Examples of biomimetic applications	Relevant citations for biomimetic applications
Morpho butterfly	Wings	Lamellar grating	Constructive interference	Angle-dependent photonic coatings: Optical biosensors, anti-counterfeiting coatings, *etc.*	Jonza et al. (1999)
Hummingbirds	Feathers	Alternating layers of keratin, air, and melanosomes			Minh et al. (2024)
Sunflowers	Petals	UV coloration	Bullseye patterns	Visual beacons, UV-reactive inks for agricultural applications	Glover and Whitney (2010)
Anchovy	Dermal tissue	Multilayer stacks of guanine crystals	Directional reflection and undulatory motion	Self-coordinated robots for exploration, search-and-rescue *etc.*	Berlinger et al. (2021)
Snapping shrimp	Claw	Grating inside the claw	Cavitation bubble	Ultrasonic lithotripsy, precision cleaning, localized drug release	Tang and Staack (2019)
Cricket	File, plectrum, tympana	Stridulation	Sound emission/reception	Compact sound localization systems, e.g., for micro-drones	Ruffier et al. (2011)
Electric eel	Electrocyte-based organs	Stack arrangement; coordinated discharge	Electrogenesis	Voltaic pile, communication systems with live fish	Volta (1800), Donati et al. (2016), Worm et al. (2017)
Mormyrid					
Shark	Ampullae of Lorenzini	Specialized electrosensory cells in the skin	Electroreception		
Ray					
Mormyrid	Tuberous organs				
Butterfly	Wings	Androconia	Pheromone release	Scent-delivery devices	Silina et al. (2017), Tillotson and Temple (2018)
Ant	Abdomen	Pheromone gland		Ant colony optimization algorithms, biosensors	Colorni et al. (1991), Dorigo and Stützle (2004), Lu and Liu (2022)
Grapefruit	Fruit	Extracellular vesicles	Intercellular exchange and communication	Drug delivery	Feng et al. (2023)
Various plants, animals	Cell membrane	Aquaporins	Selective water passage	Water filtration	Le Duc et al. (2011), Hu et al. (2012), Itoh et al. (2022)
CAM plants	Aerial organs, including leaves	Stomata	Regulation of gas exchanges	Self-humidifying membranes	Park et al. (2016)

The examples listed in this table follow their order of appearance in the review.

#### 2.1.1 Directional reflection

The physical properties of a surface, including its composition, geometry, and refractive index gradients, determine its reflective and refractive behavior. Several species have evolved body surfaces capable of reflecting light in specific directions, thereby producing structured optical signals known as directional reflection. This mechanism enables the emission of visual cues that can carry biological meaning, particularly in intraspecific communication (Vukusic and Sambles, 2003).

**FIGURE 1:**
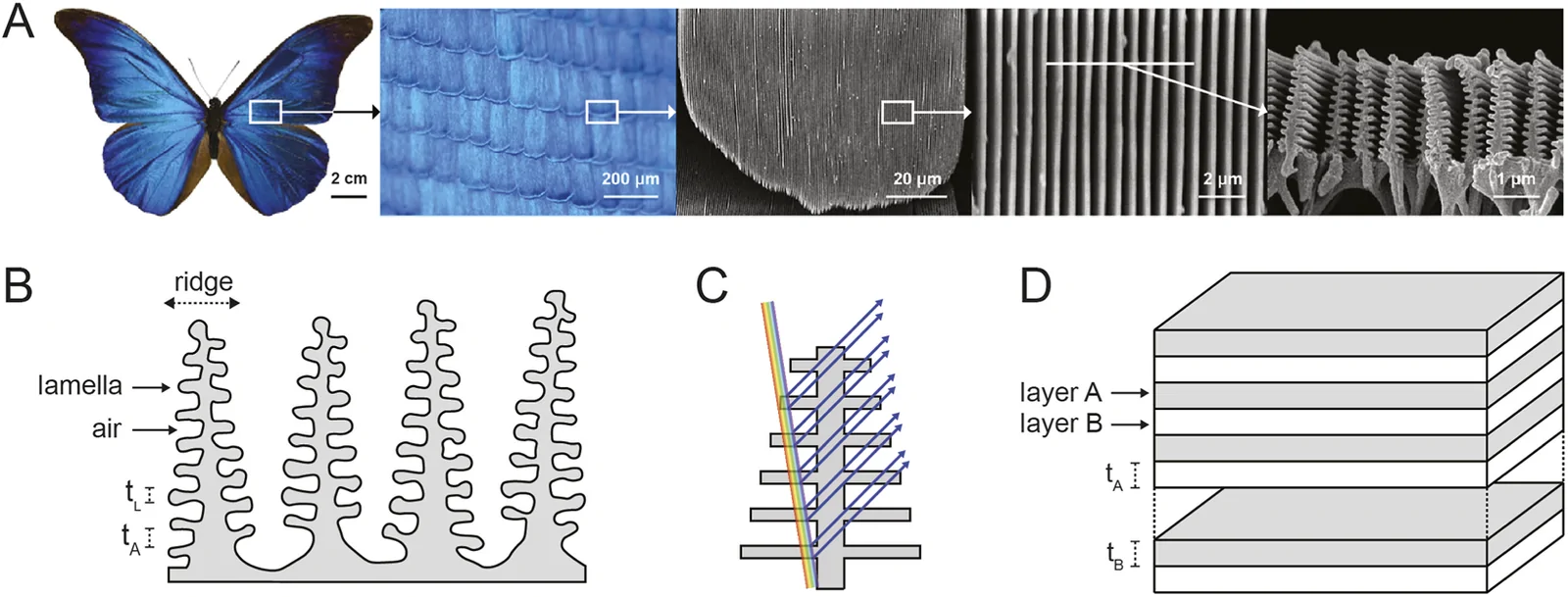
The Morpho butterfly wing color. **(A)** From left to right: dorsal view, optical microscope image, and three electron microscope images taken from *Morpho rhetenor*. Each picture shows progressively finer structural details, from the blue wings to the arrangement of their scales, the ridges at the surface of each scale, and finally a cross-section of the ridges revealing the superimposed lamellae. Photo credits: left image reproduced from (Wikipedia Contributors, 2025): licensed under CC BY-SA 4.0. All four other images (Song et al., 2017): licensed under CC BY 4.0. **(B)** Diagram showing the lamellar organization of the ridges (t_L_ and t_A_ represent the thickness of each lamella and air pocket, respectively). While the exact measurements of t_L_ and t_A_ may differ across species, the spacing between consecutive lamellae (i.e., t_L_ + t_A_) has consistently been found to be 200 nm across various *Morpho* species (Kinoshita et al., 2002; Berthier, 2010). **(C)** Despite having no blue pigment, the Morpho butterfly wings appear blue. This is due to the periodic organization formed by alternating layers of lamellae and air. This grating causes constructive interferences which reflect blue wavelengths specifically. **(D)** Diagram of the Clear-to-color security film (Jonza et al., 1999), a biomimetic invention inspired by the lamellar grating of the Morpho butterfly. The film is made of alternating layers of material A and B (of thickness t_A_ and t_B_, respectively). The characteristics of the film, such as its reflecting color and clear-to-color view angle, are controlled through the composition of materials A and B, their respective thickness, and the total number of layers reproduced from (Song et al., 2017), licensed under CC BY 4.0.

The *Morpho* butterfly provides a well-documented example. Its wing scales feature longitudinal ridges composed of superimposed lamellae with regular spacing of approximately 200 nm (Figures 1A,B). This periodic organization forms a lamellar grating that produces selective constructive interference in blue wavelengths (Ghiradella, 1991; Kinoshita et al., 2002; Berthier, 2010) (Figure 1C). The resulting reflection is strongly directional, with a peak reflectance of up to 70%, visible only under specific viewing angles. This highly anisotropic optical response functions as a sexual signal visible to conspecifics positioned favorably, while limiting detection by potential predators (Sweeney et al., 2003). Such traits illustrate a multiscale architecture which incorporates structural features operating across multiple spatial scales, whose combined interactions contribute to the overall function of the system.

The *Morpho* model has inspired numerous biomimetic photonic materials that replicate its multilayer nanostructure to control light propagation. Engineers have designed *Morpho*-like multilayer films and gratings with applications in ultrasensitive optical biosensors, where shifts in reflected color indicate molecular binding at the surface, and in anti-counterfeiting coatings that exploit angle-dependent reflectance as a hidden identifier. For instance, the *Clear-to-Colored Security Film* (Jonza et al., 1999) applies this principle of interference-based color change, remaining transparent at normal incidence but displaying a cyan hue beyond a critical viewing angle (Figure 1D).

While insects are archetypal examples, directional reflection is also widespread among vertebrates. In hummingbirds (Trochilidae family), feathers contain alternating layers of keratin, air, and melanosomes. The relative thickness, density, and alignment of these multilayers determine the interference conditions, producing iridescent colors that vary with viewing angle (Gruson et al., 2021). These visual signals play a role in species recognition and sexual display (Simpson and McGraw, 2019). The underlying mechanism (Doucet et al., 2006) has inspired angle-dependent photonic coatings and iridescent devices whose color output changes according to the observer’s position or illumination angle. Such biomimetic systems are now used in visual encryption, where information or images are encoded in angular color variations, and in context-sensitive optical displays for authentication or adaptive decoration (Minh et al., 2024).

Many examples of directional reflection can also be found in plants. On the petals of sunflowers (*Helianthus annuus*), absorbent UV patterns called bullseyes guide pollinating insects toward the reproductive organs. Invisible to the human eye, these structures are perceptible under UV vision and enhance cross-pollination efficiency (Heiser et al., 1969; Whitney et al., 2009; Glover and Whitney, 2010; Hempel De Ibarra et al., 2014). This model has inspired the development of visual beacons for agricultural drones, designed to improve the accuracy of pollination and crop monitoring systems. By mimicking the UV contrasts used by flowers to attract pollinators, these artificial markers enable drones to identify specific floral targets or detect plant reproductive zones. Similarly, UV-reactive inks reproducing these contrasts are used for functional marking in agriculture and environmental sensing (Glover and Whitney, 2010). Synthetic biology could extend this field by engineering plants with modified UV-reflective structures or controllable pigment pathways to enhance pollinator guidance or support precision agriculture. Recent advances in plant pigment engineering show that pigment biosynthetic pathways can be systematically reprogrammed using synthetic regulatory tools such as small RNAs and targeted genetic manipulation (Barrera-Rojas et al., 2025), demonstrating the feasibility of engineering visual signaling traits in plants through direct modification of pigment biosynthesis pathways.

Finally, directional reflection is not restricted to one-on-one communication: some species use this phenomenon to coordinate collective movement within large groups. For example, anchovy (*Engraulis encrasicolus*) scales are rich in guanine crystals, arranged in multilayer stacks within the dermal tissue (Denton and Land, 1971; Lafoux et al., 2023). These crystalline arrays act as biological mirrors, producing strong directional reflection when illuminated. As the fish swims, undulatory motion periodically reorients these reflective layers, generating rapid flashes of light visible at distances exceeding 2 m. These flashing signals act as synchronization cues within the fish school, allowing individuals to coordinate their orientation and spacing. Computational models show up to 94% collective alignment based solely on the optical perception of 3–5 nearby neighbors (Lafoux et al., 2023). This constitutes a systems biology framework in which behavioral dynamics emerge from simple, locally perceived optical signals within a distributed, self-organizing network.

This biological principle inspired the design of the Bluebots, a swarm of biomimetic underwater robots developed at the Wyss Institute at Harvard University (Berlinger et al., 2021). Each Bluebot is equipped with directional LEDs and cameras that replicate the optical signaling of schooling fish. By detecting and responding to light flashes from neighboring robots, the swarm achieves distributed coordination without centralized control. This system illustrates how the optical communication strategies of fish can be translated into autonomous robotic networks for collective exploration, environmental monitoring, and search-and-rescue applications. Because these robots implement decentralized rules derived from biological models, they echo systems biology insights into emergent coordination.

Altogether, directional reflection exemplifies how biological surfaces encode and transmit information through light–matter interactions. By reproducing the multiscale architectures that govern natural reflectance multilayered scales, such as refractive index contrasts, or crystalline arrays, biomimetic engineering enables the creation of optical communication interfaces capable of controlled light emission, dynamic information encoding, and collective signaling.

#### 2.1.2 Mechanical propagation

Beyond optical communication, many species also rely on mechanical signaling to convey information through their environment. These signals arise from the interaction between the mechanical properties of biological surfaces and the physical characteristics of the surrounding medium, whether water, air, or a solid substrate (Cocroft and Rodríguez, 2005). From a systems biology perspective, such signals can be seen as components of integrated communication networks in which physical, sensory, and behavioral elements jointly determine information flow.

Among the most remarkable cases are systems that exploit fluid-induced mechanical disturbances. High-speed imaging of the snapping shrimp (*Alpheus heterochaelis*) has shown that the radial arrangement of ridges inside its claw governs the formation, position, and lifetime of cavitation bubbles (Schein, 1977; Versluis et al., 2000; Pandit et al., 2025). During rapid closure, the claw ejects a jet of water exceeding 6 m.s^-1^, creating a cavitation bubble whose collapse produces a shock wave and flash of light (Herberholz and Schmitz, 1999). Inspired by this mechanism, engineers have reproduced the claw’s microtopography on textured surfaces and piezoelectric emitters, enabling controlled nucleation and asymmetric collapse of bubbles at defined sites. These biomimetic microcavitation systems generate directional microjets and pulsed shock waves, now exploited in non-invasive ultrasonic lithotripsy, precision cleaning of optical and electronic components, and localized drug release in microfluidic devices (Tang and Staack, 2019). Although this approach belongs to the field of engineering biology rather than biomimetic surface design, a recent study has demonstrated that genetically encoded gas vesicles can be engineered to produce controlled acoustic responses and remotely triggered mechanical effects in living tissues (Bar-Zion et al., 2021). While not directly reproducing the cavitation mechanism of the snapping shrimp, this proof-of-concept system illustrates how biological structures may eventually be engineered for targeted mechanical actuation.

Other organisms rely instead on surface-borne vibrations to transmit information. In crickets (Gryllidae family), sound generation through stridulation arises when a toothed appendix, called the file, scrapes against a rigid plectrum, setting a thin membrane into resonance (Bennet-Clark, 1999; Stritih-Peljhan and Virant-Doberlet, 2021). Signal reception occurs through a pair of tympana on the forelegs, coupled by an internal tracheal network that enables phase-difference detection (Bennet-Clark, 1999). This multi-component sensory architecture exemplifies a systems biology framework, in which mechanical structures, air-flow pathways, and neural processing act together as an integrated directional-hearing system. This bilateral acoustic system has inspired the design of biomimetic micro-electro-mechanical systems that achieve passive sound localization through interconnected resonant membranes, offering compact, low-energy orientation modules for micro-drones (Ruffier et al., 2011). Synthetic biology could further expand this field by engineering living mechanosensory tissues or genetically encoded resonant structures capable of modulating vibration-based communication (Garamella et al., 2019).

#### 2.1.3 Electrogenesis and electroreception

Beyond the mechanical realm, some species have evolved the ability to encode and transmit information through electric fields rather than physical motion. This shift from surface-mediated mechanical vibrations to bioelectric signaling marks another level of sophistication in biological communication, where the surface itself becomes both the emitter and receiver of electrical information.

Electrogenesis and electroreception are defined as the ability for some animals to generate and perceive electrical fields, respectively (Hopkins, 1999; Crampton, 2019). Because aquatic environments efficiently transmit weak electric fields, most species exhibiting these traits are found in freshwater or marine ecosystems and are collectively referred to as electric fish (Heiligenberg and Bastian, 1984; Caputi, 2023).

Among them, the electric eel (*Electrophorus electricus*), the electric rays (*Torpediniformes* order), and the mormyrids (Mormyridae family) display highly specialized electrocyte-based organs (Heiligenberg and Bastian, 1984; Caputi, 2023). These cells, derived from muscle fibers, are arranged in stacks under the skin along the trunk or tail, where their coordinated discharge produces a cumulative electric potential (Hopkins, 1999; Crampton, 2019). Depending on the species, discharges can be brief pulses or continuous sinusoidal waves, with frequencies modulated by social context, such as aggression, courtship, or orientation, enabling an electrical language based on signal variation (Hopkins, 1999; Crampton, 2019). Historically, the principle of these biological batteries inspired Alessandro Volta, who explicitly described his Voltaic pile as an “artificial electric organ” (Volta, 1800).

Conversely, electroreception depends on specialized receptors distributed across the skin, such as the ampullae of Lorenzini found in sharks and rays (*Elasmobranchii* subclass), or similar tuberous organs in weakly electric fish such as mormyrids. These sensory structures detect electric field gradients of only a few microvolts with remarkable spatial and temporal resolution, enabling both prey localization and social communication (Bennett, 1971; Bullock, 1982; Caputi, 2023).

The dual function of generation and detection forms the basis of electrocommunication, a form of contactless dialogue mediated by the surface of the skin (Hopkins, 1999; Crampton, 2019). This phenomenon has inspired the development of bioinspired robotic fish, capable of reproducing the electric signaling patterns of real species. Covered with soft polymeric skins embedded with distributed electrode arrays, these robots can emit coded pulses that elicit social or group behaviors from live fish (Donati et al., 2016; Worm et al., 2017). Such systems illustrate how electrically active biological surfaces can inform the design of non-invasive, adaptive communication interfaces capable of exchanging information within aquatic or biological environments without disrupting their integrity (Caputi, 2017).

In addition to these biomimetic solutions, synthetic biology is beginning to explore bioelectric control circuits, through the engineering of gene networks and ion-channel-based actuators that could eventually enable engineered cells to generate programmable electrical signals analogous to those used by electric fish (Mansouri and Fussenegger, 2025).

#### 2.1.4 Chemical communication

Chemical communication exemplifies how biological surfaces function as dynamic interfaces for the exchange of information. Far from being passive boundaries, these surfaces organize, modulate, and transmit molecular signals through finely tuned physical and chemical properties. By adjusting parameters such as volatility, polarity, and surface topography, organisms encode social or environmental information across space and time. From a systems biology perspective, these processes reflect coordinated interactions across molecular, structural, and behavioral levels, forming integrated networks with emergent coordination rather than isolated signaling events (Michailidu et al., 2025).

In insects, the release of pheromones often relies on cuticular microstructures that regulate both emission and directionality (Wyatt, 2014; Renou and Anton, 2020). The androconia scales of butterflies, for instance, act as diffusive matrices whose microscopic geometry amplifies the effective surface area while wing movements actively shape the air flow, orienting the chemical plume toward a receiver (Grula et al., 1980; Nieberding et al., 2008; Ehlers and Schulz, 2023). This biological control of spatial dispersion has directly inspired smart olfactory systems designed to reproduce adaptive and directional release. The company eScent, for example, developed wearable scent-delivery devices that combine microencapsulation with environment-responsive diffusion control, emulating the context-dependent pheromone signaling strategies found in nature (Silina et al., 2017; Tillotson and Temple, 2018). However, commercial implementations remain limited in scope and validation.

**FIGURE 2:**
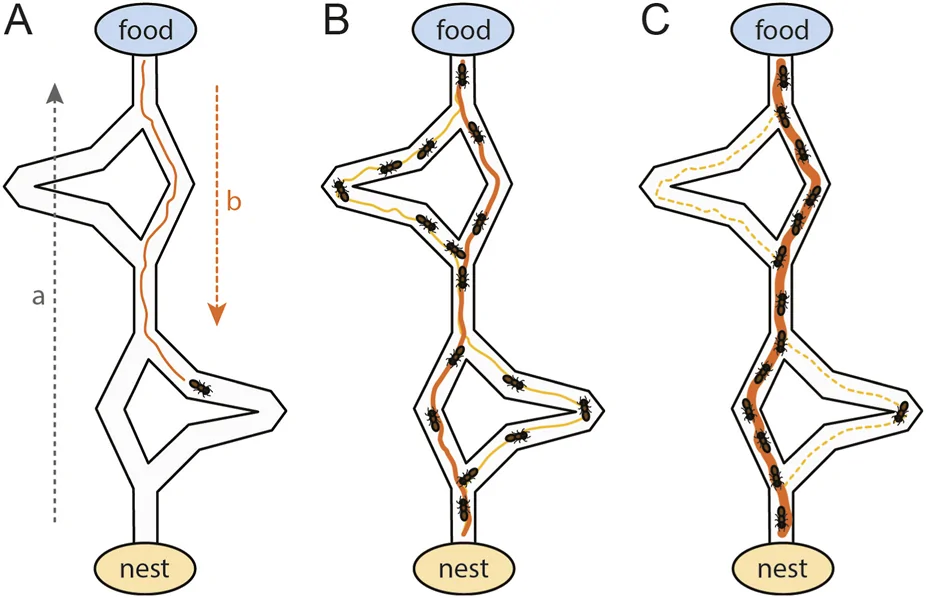
Principle of the Ant Colony Optimization algorithm. Adapted from (Liu et al., 2021) licensed under CC BY 4.0. **(A)** An ant travels from its nest to a food source (arrow a) and then returns to the nest (arrow b). During the return trip, the ant deposits a pheromone trail, with a concentration proportional to the quality of the path (i.e., shorter paths receive a higher pheromone concentration). **(B)** As more ants travel between the nest and the food source, all possible paths become marked with pheromones. **(C)** The shortest path accumulates the highest pheromone concentration and becomes the preferred route. In contrast, longer paths retain weaker pheromone levels. Over time, these weaker trails diminish due to evaporation, reinforcing the dominance of the shortest path.

At the collective scale, other species use chemical gradients as persistent markers to coordinate group behavior. Pheromone trails deposited on substrates by foraging ants, for example, can persist for minutes to hours, forming chemical fields that encode spatial memory and trigger specific social responses (Jackson and Ratnieks, 2006; David Morgan, 2009). The logic underlying this distributed, surface-mediated information storage has inspired the Ant Colony Optimization algorithms (Colorni et al., 1991; Dorigo and Stützle, 2004), in which the dynamics of chemical trails are abstracted into computational processes optimizing communication and movement within multi-agent systems (Figure 2). These emergent, feedback-driven patterns are emblematic of systems biology, where collective behavior arises from simple local rules executed across interacting agents.

The same principles of distributed chemical sensing and feedback have guided the development of bioinspired interfaces. Recent studies on insect olfactory systems have led to surface-based biosensors capable of detecting volatile compounds through integrated biological receptors or antenna-like microstructures, reproducing the sensitivity and selectivity of natural chemoreception (Lu and Liu, 2022). Although these devices remain biomimetic, advances in synthetic biology are beginning to engineer cells and tissues capable of producing, sensing, or processing chemical signals, leaving room for future innovations (Moghimianavval et al., 2025).

### 2.2 Exchanges

Exchanges are a key feature of communication but are also implied in various physiological and biological mechanisms. They can be defined as the passage of biologically relevant molecules across cells and tissues (De Luis et al., 2021). Here we will focus on three exchange mechanisms which have given rise to biomimetic and bioinspired technologies: extracellular vesicles (EVs), aquaporins, and plants’ stomata. Understanding these systems helps reveal the regulatory networks and transport dynamics that govern these molecular exchanges, while synthetic approaches enable the engineering of tailored channels, vesicles, or responsive interfaces that emulate and expand these natural mechanisms (Table 1).

#### 2.2.1 Extracellular vesicles

EVs are small, membrane-bound particles released by cells into the extracellular environment. They play important roles in intercellular exchanges by transporting proteins, lipids, and nucleic acids between cells. Initially considered as cellular debris, EVs are now recognized as key mediators in physiological processes and disease, including cancer, immune responses, and tissue regeneration (Dixson et al., 2023). From a systems biology perspective, EVs function as nodes within distributed signaling networks, integrating molecular cargo, cellular states, and tissue-level responses into coordinated biological communication. In recent years, EVs have inspired various innovative drug delivery technologies (EL Andaloussi et al., 2013; Ohno et al., 2016; Niu et al., 2021; Feng et al., 2023).

One example can be found in the treatment of vascular calcification (VC). VC is the abnormal deposition of calcium phosphate minerals in the walls of blood vessels, leading to stiffness, reduced elasticity, and impaired cardiovascular function. While treatments exist, they bear severe side effects, which are exacerbated by the fact that, to reach and penetrate the calcified tissues, those drugs need to be injected at high doses (Lanzer et al., 2021).

Researchers have designed a biomimetic nanocarrier derived from grapefruit (*Citrus x paradisi*) EVs, for the targeted delivery of VC drugs. Compared to animal-based EVs, EVs extracted from plants bear several advantages, namely, higher yields, lower immunogenicity, and cheaper production costs. In addition, the EVs of some plants, such as grapefruit, have natural anti-inflammatory properties, which the researchers wanted to retain. The engineered nanocarrier showed enhanced uptake by calcified vascular smooth muscle cells and effectively inhibited their calcification. Importantly, the treatment showed no systemic toxicity, highlighting its potential as a safe and effective vessel for delivering VC treatments (Feng et al., 2023). While these vesicles are engineered only at the material level, parallel advances in synthetic biology are beginning to develop engineered or hybrid EV-like systems capable of producing or packaging defined molecular cargos, pointing toward future interfaces between biological vesicle signaling and programmable EV-mimetic technologies (Kelwick et al., 2024). However, particular attention must be paid to their biodistribution and eventual metabolization, as well as the homogeneity of their cargo between production lots.

Note that, as EVs play a key role in cell communication, they could have been discussed earlier in this review, in the dedicated subpart “Communication”. Here we chose to emphasize the exchange mechanisms with regards to biomimicry.

#### 2.2.2 Aquaporin

Aquaporins are specialized membrane proteins that facilitate the transport of water molecules across cell membranes. They are found in both plants and animals and play a crucial role in maintaining water balance by regulating osmotic gradients. Structurally, all aquaporins share a conserved architecture: six transmembrane helical segments and two short helices flanking cytoplasmic and extracellular vestibules, connected by a narrow aqueous pore. Water selectivity during transport is governed by steric hindrance and electrostatic interactions within the pore, shaped by the conformation of the protein subunits (Kruse et al., 2006; Verkman, 2013; Roche and Törnroth-Horsefield, 2017).

Researchers have developed multiple generations of synthetic aquaporins using compounds such as imidazole-quartets (Le Duc et al., 2011) and hydrazide pillars (Hu et al., 2012). These synthetic channels aim to replicate the selective water transport properties of natural aquaporins, which can be then applied to water purification. By mimicking the aquaporin’s ability to restrict ion flow while allowing water passage, these systems offer enhanced filtration performance (Le Duc et al., 2011; Hu et al., 2012; Itoh et al., 2022). Although these constructs are non-living, they represent an approach adjacent to synthetic biology, in which biological transport functions are recreated through engineered molecular architectures, echoing the design principles of synthetic biological membranes.

However, the first generation of synthetic aquaporins exhibited lower water permeability compared to the native protein, primarily due to hydrogen bonding interactions that disrupted water flow (Song and Kumar, 2022). To address this limitation, second-generation designs incorporated hydrophobic groups into the pore structure, which improved water permeability but compromised ion selectivity through cation–π interactions (Song and Kumar, 2022). These new designs offer promising avenues for next-generation filtration technologies (Itoh et al., 2022; Sato et al., 2022; Song and Kumar, 2022). This iterative optimization process parallels synthetic biology design cycles: defining function, testing engineered variants, and refining properties toward modular, stimuli-responsive membrane behavior.

#### 2.2.3 Stomata

While water control within a cell is a key condition of life, multicellular organisms also need to tune water exchanges through their tissues. Plants possess a dedicated structure called stoma to regulate these exchanges (Falquetto-Gomes et al., 2024). Stomata are pores found in the epidermis of various organs including the leaves. By finely controlling their opening and closing, plants regulate gas exchanges, including water, between the inside and the outside of their organs (Falquetto-Gomes et al., 2024). This regulation integrates environmental sensing, hormonal signaling, and tissue-level mechanics, making stomata a prime example of a systems biology process where emergent behavior results from multilayered biological control circuits.

**FIGURE 3:**
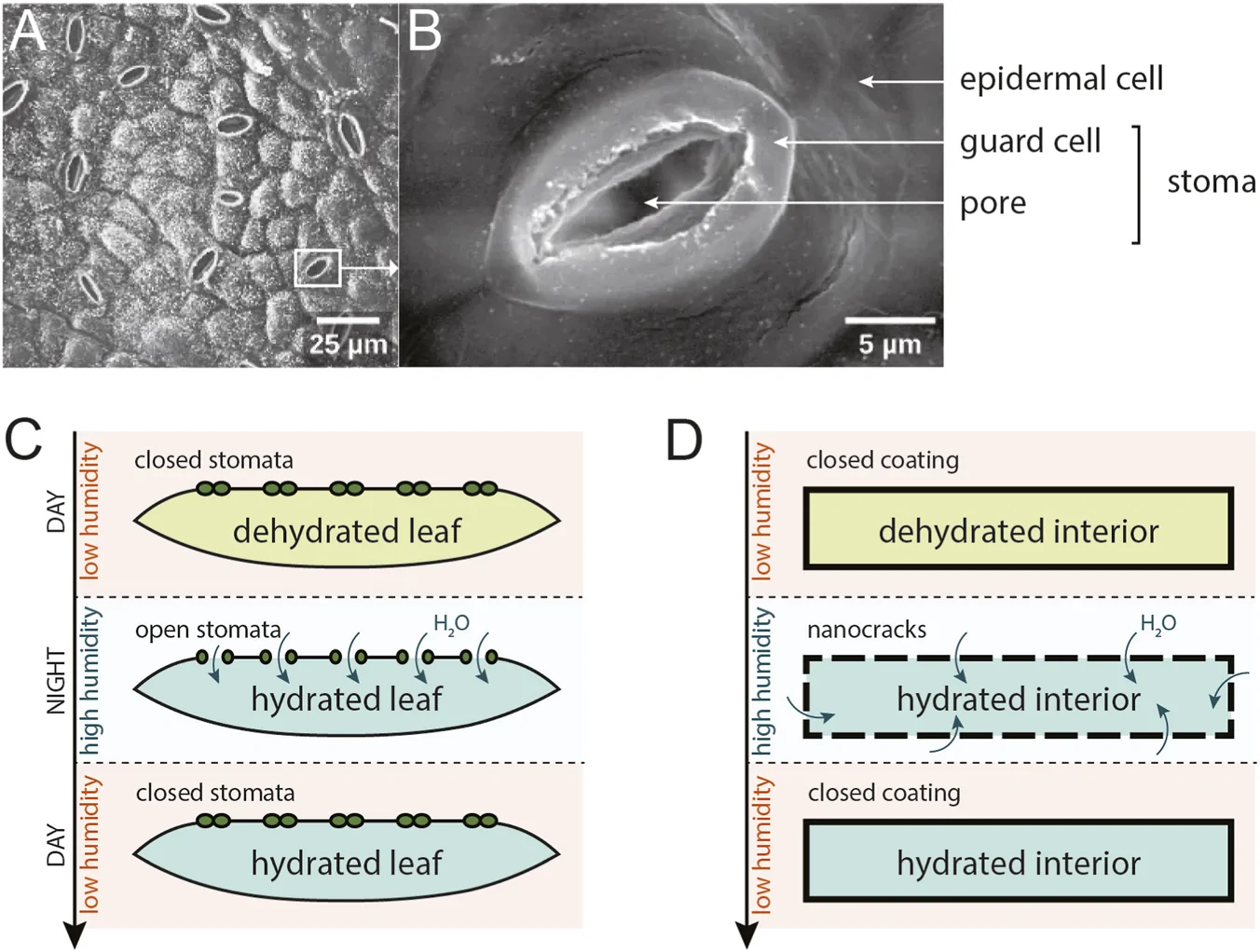
The stomata of CAM plants. **(A)** Electron micrograph of a sumac (*Rhus glabra*) leaf. Stomata form pores in the epidermis. Photo credit reproduced from (Rye et al., 2016): licensed under CC BY 4.0. **(B)** Close-up view of a stoma. Each pore is surrounded by a pair of guard cells. Photo credit reproduced from (Rye et al., 2016): licensed under CC BY 4.0. **(C)** Stomatal opening and closing cycle in CAM plants. Stomata open at night, when temperatures are lower and humidity is higher, reducing water loss and improving water-use efficiency. **(D)** Self-humidifying membrane inspired by CAM plants. The hydrophobic coating opens under humid conditions through the formation of nanocracks.

In *Crassulacean Acid Metabolism* (CAM) plants including cacti, which are usually found in dry environments such as deserts, the stomata close during the day and open at night, when the temperature drops and the humidity rises (Figures 3A–C). This mechanism allows CAM plants to perform gas exchange only at night, which lowers water loss. Overall, CAM stomatal behavior embodies systems-level adaptation, linking circadian rhythms, metabolic states, and environmental cues into a unified regulatory system (Tan and Chen, 2023).

Park *et al.* used the same stomatal properties in 2016 to design a biomimetic, self-humidifying membrane. A thin hydrophobic membrane is placed over narrow water channels called nanocracks, which open under humid conditions, allowing regulation of water exchange while preventing the hydrophobic layer from hindering ion co-transport with water (Figure 3D). Such surfaces could be used in an array of applications, from chemical batteries to water purifying systems (Park et al., 2016).

### 2.3 Design principles emerging from communication and exchange through biological surfaces

Taken together, the examples discussed in this section reveal a striking convergence toward a limited number of transferable design principles that extend beyond individual biological systems. The photonic structures of *Morpho* butterflies (Kinoshita et al., 2002), hummingbirds (Doucet et al., 2006), sunflowers (Glover and Whitney, 2010), and anchovies (Lafoux et al., 2023) exemplify how multiscale and graded architectures can transform nanoscale organization into organism-level communication functions. These same examples illustrate anisotropy and directionality, as optical signals are transmitted only under specific viewing conditions to control information flow. In anchovy schools (Lafoux et al., 2023) and ant pheromone trails (David Morgan, 2009), collective signaling arises from local interactions among individuals, demonstrating emergent behavior and decentralized information processing. Communication systems based on electric fish such as electric eels and mormyrids (Caputi, 2023), as well as mechanically mediated signaling in snapping shrimp (Pandit et al., 2025) and crickets (Stritih-Peljhan and Virant-Doberlet, 2021), further show how biological surfaces integrate physical structures and sensory networks to generate coordinated responses. Likewise, stomata in CAM plants (Tan and Chen, 2023) and aquaporins (Verkman, 2013) illustrate stimuli-responsiveness and the importance of trade-offs and Pareto fronts, balancing water conservation against gas exchange or permeability against selectivity. Finally, extracellular vesicles (Dixson et al., 2023) highlight the value of modularity, where layered and compartmentalized systems achieve controlled exchange while maintaining scalability and robustness. These recurring principles suggest that future communication and exchange interfaces could benefit not only from reproducing individual biological models, but also from translating the underlying organizational rules that govern information transfer and molecular transport across living surfaces.

## 3 Defense

In addition to regulating exchanges with the outside environment, biological surfaces ensure its organism’s defense against external aggressions (Kikuchi et al., 2023). To escape or resist predator attacks, organisms have developed a variety of strategies, including the use of decoys, physical barriers, and biological or biochemical defenses, all of which have given rise to biomimetic and bioinspired applications. Systems biology helps elucidate the regulatory pathways and multiscale interactions that shape these defensive surface traits, while synthetic biology enables the engineering of living or hybrid systems capable of reproducing or enhancing such protective functions (Table 2).

**TABLE 2:** Biological examples and biomimetic applications discussed in this review, related to defense.

Species	Organ/body part	Mechanism/structure	Biological phenomenon	Examples of biomimetic applications	Relevant citations for biomimetic applications
Cephalopods	Skin	Platelets of highly reflective reflectins	Background matching	Dynamically-changing materials, adaptive implant coatings	Lychko et al. (2025)
Chameleon		Nanocrystals		Soft robot camouflage	Kim et al. (2021)
Chameleon prawn					
Razorbill	Feathers	Two-toned body color	Countershading	Camouflage of military vehicles, equipment, and personnel	Elias (2008), Behrens (2009), Cuthill et al. (2016), Ryan et al. (2024)
Velvet belly lanternshark	Underside skin	Luminescent underside			
Moth	Eyes	Nipple arrays	Anti-reflection	Solar-powered medical devices, solar energy harvesting, medical imaging, sensing technologies	Hong et al. (2009), Isakov et al. (2020), Jiao et al. (2023), Oliva et al. (2022), Yang et al. (2017)
Leafhopper	Wings	Nano-cone arrays; brochosome coating			
Dragonfly		Nano-cone arrays			
Butterfly					
Zebra	Body surface	Contrasting patterns	Color disruption and motion dazzle	Dazzle camouflage (world war I), camouflage textiles	Thayer (1923), Motta and Didero (2024)
Tiger					
Shore crab	Carapace				
Various animals	Body surface	Bright colors; striking patterns	Aposematism	Warning signage	Saito (2011)
Peacock butterfly		Colors and patterns mimicking other species	Batesian mimicry		
Hoverfly					
Fish	Skin	Arrangement of scales	Hardness and flexibility	Flexible armors, stretchable electronics, soft robotic skins	Chintapalli et al. (2014), Islam et al. (2021), Tatari et al. (2023)
Mollusk	Shell	Nacre (mother-of-pearl)	Hardness and biocompatibility	Biomedical surface coatings (orthopedic, dental implants)	Tan et al. (2019), Tabrizian et al. (2023)
Pangolin	Body surface	Bony plates	Hardness and thermal resistance	Protective coatings, thermal armors	Prötsch et al. (2025), Shah et al. (2024), Le and Goo (2019)
Arapaima fish					
Armadillo					
Cephalopods	Skin	Muscle fiber contraction	Responsive stiffness	Metamaterials (adjustable stiffness, damping, and energy dissipation)	Fei et al. (2021), Wang et al. (2025), Yan et al. (2024)
Various plants	Stem	Touch hypersensitivity	Responsive folding		
Insects	Exoskeleton	Folding joints	Stiffness and flexibility	Metamaterials, soft robotic joints	Kaufmann and Li (2020)
Lotus	Leaf	Waxy cuticle	Self-healing when scratched	Self-healing surfaces	Hu et al. (2024)
Cicada	Wings	Nanopillars	Stretching, rupturing of bacterial membrane	Antibacterial surfaces	Jin et al. (2024), Zhang et al. (2022)
Dragonfly					
Rose	Petals	Micro-sized asperities	Superhydrophobic surface, impairing bacterial settlement and development		
Gecko	Skin	Hair-like structures			
Various animals	Body surface	Mucin-rich mucus	Release of antimicrobial compounds on the whole surface	Antibacterial coatings for implants	Tallet et al. (2021)
Tomato	Fruit skin	Cutin-derived oligomers		Antimicrobial-releasing surfaces	Wang et al. (2020), Zhuk et al. (2014)
Various animals	Surface of various organs	Various antimicrobial substances	Release of antimicrobial compounds at the site of infection		
Various marine algae and corals	Body surface	ROS		Catheters, wound dressings, antimicrobial photodynamic therapy	Omidiyan et al. (2024), Li et al. (2023), Melo et al. (2025)

The examples listed in this table follow their order of appearance in the review.

### 3.1 Decoys

Decoy strategies are achieved by either making the organism undetectable in its environment through camouflage (Wallace, 2010), or, conversely, warning others of the harm it may cause to predators, displaying vibrant colors, a phenomenon called aposematism (Rojas et al., 2015).

#### 3.1.1 Camouflage

Camouflage is the use of disguise to either make oneself hard to see or appear as something else. It often depends on the properties of biological surfaces, especially their structure, texture, pigmentation, and interaction with light (Poulton, 1890; Thayer, 1896; 1923; Thayer and Thayer, 1909; Cuthill, 2019). One of the most widespread camouflage strategies is background matching, where the surface color and pattern of an organism blend into its surrounding environment (Cuthill et al., 2005; Merilaita and Lind, 2005).

Cephalopods such as squids, cuttlefish, and octopuses are renowned for their background matching abilities. They can rapidly change the color, contrast, and pattern of their skin to match their surroundings (Chiao et al., 2011). This camouflage is achieved through a sophisticated multilayered skin structure composed of chromatophores, leucophores, and iridophores, containing membrane-enclosed platelets of highly reflective reflectins (Tao et al., 2010; Levenson et al., 2017). This multicomponent system exemplifies systems level integration of neural control, pigment cells, and structural optics, with emergent camouflage behavior arising from interactions across scales.

Recent engineering studies created reflectin-based materials, inspired by the optical properties of cephalopod skin (Lychko et al., 2025). These materials possess unique optical properties, such as the ability to change color dynamically and reversibly, in response to stimuli such as humidity, acid vapors or even light. They can form films with angle-dependent reflectance or colored multiscale structures via controlled self-assembly. In addition, they show good proton conductivity and are biocompatible, supporting their use in biomedical applications such as adaptive implant coatings.

**FIGURE 4:**
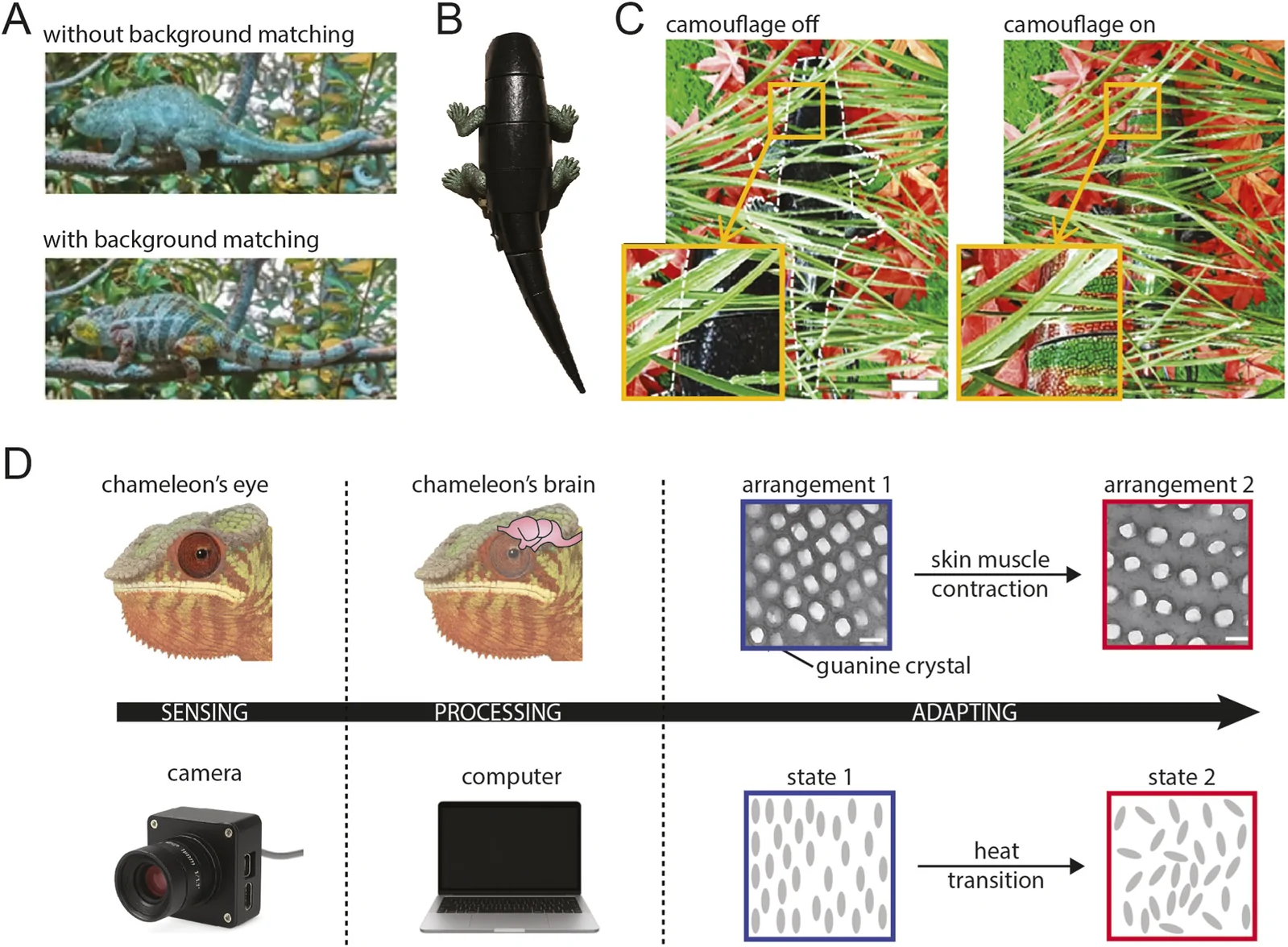
Chameleon’s background matching. **(A)** The same chameleon before (top) and after (bottom) changing the color of its skin to match its background. Photo credit reproduced from (Kim et al., 2021): licensed under CC BY 4.0. **(B)** Chameleon-inspired robot. The robot is covered with a soft surface that changes color in response to its surroundings. Photo credit reproduced from (Kim et al., 2021): licensed under CC BY 4.0. **(C)** Robot placed in a colorful environment, shown before (left) and after (right) background matching. Photo credit reproduced from (Kim et al., 2021): licensed under CC BY 4.0. **(D)** Top: As the chameleon observes its surroundings, it determines whether and how to change color. It adapts by contracting specialized skin cells, which alters the spacing between guanine nanocrystals embedded in the dermis. The resulting lattice structure controls light reflection, leading to a change in skin color. Bottom: The chameleon-inspired robot is covered with soft, camouflage-capable surfaces composed of a layer of thermochromic liquid crystals over patterned silver nanowire heaters. A camera acts as the chameleon’s eye and captures the surroundings. The images are processed computationally to determine the appropriate color response. Localized heating then induces a phase transition in the liquid crystals, altering their optical properties and thus their color. Adapted from (Teyssier et al., 2015) licensed under CC BY 4.0.

In the same way, the skin of chameleons (Chamaeleonidae family) contains guanine nanocrystals that reflect different wavelengths of light depending on their spacing, allowing the animal to rapidly change color and adapt to its surroundings (Figure 4) (Stuart-Fox et al., 2008). Chameleon prawns (*Hippolyte varians)*, which are named after the color-changing reptile, share the same camouflage mechanism (Green et al., 2019). These color change systems operate through integrated physiological, structural, and environmental signaling pathways, aligning with systems biology notions of multiscale regulation. Taking inspiration from these nanocrystals, researchers have developed soft robots camouflaging surfaces using thermochromic liquid crystals and patterned silver nanowire heaters, combined with sensors to match their surroundings (Figure 4D) (Kim et al., 2021).

Countershading and counterillumination are two other widespread camouflage techniques, in which the animal conceals its self-shadow by matching skin color or bioluminescence. For example, the Razorbill (*Alca torda*), among other seabirds, uses two-toned countershading: its dark dorsal side blends with the darker depths when viewed from above, while its lighter ventral side matches the brighter, sunlit surface when seen from below (Cairns, 1986). In the same way, the velvet belly lanternshark (*Etmopterus spinax*), found in Norwegian fjords, produces light on its underside to match the intensity of downwelling ambient light (Claes et al., 2010). This adaptation effectively erases their silhouette when viewed from below, helping it to remain hidden from predators and prey (Johnsen et al., 2004; Mullan et al., 2023). Countershading and counterillumination demonstrate systems biology principles, involving a multiscale approach (i.e., sensory perception, environmental light modeling, physiological light regulation, and ecological feedback) with predators.

Both of those strategies have inspired biomimetic innovations in stealth technologies. Military prototypes such as submarines, aircraft, and drones have incorporated design principles from countershading and counterillumination to reduce visual detection in different operational environments (Elias, 2008; Behrens, 2009; Cuthill et al., 2016). Similarly, research on counterillumination mechanisms in marine animals could inform the development of light-emitting wetsuits or surfboard technologies that help surfers minimize their silhouette and reduce the risk of shark attacks (Ryan et al., 2024).

Anti-reflective strategies are also being used to protect organisms from potential predators’ attacks. For instance, nanostructures such as the hexagonally arranged nipple arrays on moth (*Heterocera* suborder) eyes and the nano-cones present on leafhopper (*Cicadella* genus), dragonfly (*Anisoptera* infraorder) and butterfly (*Papilionoidea* superfamily) wings prevent light reflection (Yoshida et al., 1997; Hooper et al., 2006; Sun et al., 2011; Chung et al., 2012; Dellieu et al., 2014). Those structures create a gradual transition of the refractive index, reducing reflectivity and making prey less visible to predators by deleting parasitic reflections. These anti-reflective mechanisms reflect systems-level optimization between surface nanostructure, incident light properties, and predator visual perception, creating directional surfaces.

Inspired by this natural design, similar surface patterns have inspired antireflective coatings on glass, lenses, screens, and solar panels (Hong et al., 2009; Isakov et al., 2020; Jiao et al., 2023). Similar nanostructures are also applied in solar-powered subcutaneous medical devices, where minimizing light reflection is essential for efficient energy absorption (Oliva et al., 2022). Additionally, the synthetic reproduction of natural nanostructures such as leafhoppers’ brochosomes (buckyball-shaped coating proteins reducing visual detectability by predators) (Stevens and Merilaita, 2009; Rakitov and Gorb, 2013; Wu et al., 2025) holds promise for various innovative applications (Yang et al., 2017). For instance, the low reflectance of synthetic brochosome-like surfaces could improve the efficiency of solar cells for energy harvesting. In imaging systems, the same materials could be used as anti-glare coatings on optical lenses. To our knowledge, these applications are yet to be explored.

Finally, various animals rely on color disruption, or motion dazzle, where contrasting patterns on the body disrupt the visual outline of an organism (Cuthill et al., 2005; Price et al., 2019). Zebras (*Equus hippotigris* subgenus) use this technique (How and Zanker, 2014; Caro and Stankowich, 2015), as well as tigers (*Panthera tigris*) (Fennell et al., 2019), and the shore crab *Carcinus maenas* (Price et al., 2019), to name a few. Motion dazzle illustrates systems biology principles since its effectiveness depends on dynamic interactions between the prey’s surface pattern, motion trajectory, and predator visual processing mechanisms.

Engineers have translated motion dazzle into textile designs such as modern military camouflage patterns (Talas et al., 2017). During World War I, dazzle camouflage involved painting allied ships with bold geometric patterns to disrupt the enemy submariner’s ability to visually estimate distance and orientation–a concept first invented by British artist Norman Wilkinson (Thayer, 1923; Santer, 2013; Meese and Strong, 2025). Similarly, startups like capable.design are working on creating textiles with patterns that would confuse facial recognition decoders using artificial intelligence, camouflaging the wearer to protect its biometric data (Motta and Didero, 2024).

#### 3.1.2 Aposematism and Batesian mimicry

In contrast to camouflage, aposematism refers to the display of highly conspicuous visual cues such as bright colors or striking patterns, or even recognizable sounds or smells to signal unpalatability or toxicity (Rojas et al., 2015; Barnett et al., 2016; Caro and Ruxton, 2019). This survival mechanism aims at deterring predators by warning them of potential danger. It is commonly seen in insects, mollusks, reptiles, amphibians, fish, and mammals (Blount et al., 2008). From a systems biology perspective, aposematism represents a multilayered communication system involving the prey’s visual or chemical signal production, predator sensory processing, learning dynamics, and ecological feedback loops that reinforce the stability of the signaling strategy within a community.

Some species even employ visual threatening strategies that mimic predator eyes or threatening features, rather than directly signaling toxicity, a strategy called Batesian mimicry. For example, butterfly species such as the peacock butterfly (*Aglais io*) display large eyespots on their wings, which can deter attacks by simulating the eyes of larger predators (Olofsson et al., 2012). Similarly, hoverflies (Syrphidae family), small and harmless pollinating flies without any stingers, imitate the yellow and black colored patterns of wasps, making them appear dangerous to the eyes of predators (Leavey et al., 2021). This mimicry system exemplifies the key systems biology principle of emergent coordination.

So far, few biomimetic and bioinspired applications take root directly in aposematism. However, the blue Morpho iridescence, for example, has inspired highly reflective and anti-fading paints, cosmetics and textiles (Saito, 2011). In the future, we may see the rise of other such innovations, such as effective warning signage inspired by aposematic patterns (Saito, 2011). Following the same logic, synthetic biology could enable the creation of programmable living warning surfaces, where engineered microorganisms or cells are designed to produce vivid aposematic pigments, fluorescence, or chemical deterrents in response to defined environmental cues (Cai et al., 2025). Such systems could form adaptive biosafety coatings that visibly signal contamination, pathogen presence, chemical exposure or structural degradation, extending aposematic logic into engineered living designs.

### 3.2 Physical and mechanical barriers

While camouflage and aposematism rely on visual deception, many organisms complement these strategies with physical and mechanical barriers. These systems act at the structural level, using surface architecture, material gradients, and spatial organization to absorb, redirect, or withstand mechanical stress. Such natural strategies are now inspiring the development of high-performance systems for use in soft robotics, biomedical devices, and body protection systems (Wegst et al., 2015).

#### 3.2.1 Passive barriers

Most multicellular organisms develop a body covering, aimed at containing the inner tissues and regulating exchanges with the outside. Body covering often serves various other functions, such as forming a passive barrier which prevents outside aggressions from degrading inner tissues (Turksen, 2017). Many species have developed elaborate protective structures within their body covering, which researchers have mimicked to create durable surfaces (Pantano and Baiamonte, 2025).

One example is found in fish scales. Biomimetic flexible armor systems have been developed by replicating the multiscale structure of these natural surfaces, which combine local stiffness with global flexibility. In one study, researchers engraved hexagonal rigid segments into a glass plate embedded in a soft substrate mimicking connective tissue, achieving up to a 70% increase in puncture resistance (Chintapalli et al., 2014). This graded architecture illustrates systems-level mechanical organization, where tissue-scale flexibility emerges from interactions among structural layers and material gradients. Building on this principle, additive manufacturing has recently been employed to recreate overlapping scale architectures in lightweight composites, paving the way for a new generation of wearable armor that combines high impact resistance with flexibility (Islam et al., 2021). Another example is found in the development of elastomeric materials with engineered scales whose orientation and spacing directly influence mechanical behavior (Tatari et al., 2023). These engineered surfaces replicate system-level functional coupling between microstructural patterning and global mechanical response.

Similarly, nacre (mother-of-pearl) has inspired the design of biomedical surface coatings and composite materials that replicate its multiscale structure, providing a remarkable combination of toughness, strength, and biocompatibility. Recent advances in nacre-mimetic calcium phosphate–polymer composites have demonstrated mechanical behavior closer to that of natural bone, due to the integration of organic–inorganic interfaces and controlled nanoscale layering (Tan et al., 2019). Nacre itself is an archetypal systems biology material, where nanoscale layering and interfacial chemistry interact to produce emergent toughness unachievable by single components alone, a prime example of graded architecture. These nacre-inspired biomaterials are currently under investigation for orthopedic and dental implant applications, offering more durable and biologically integrated alternatives to traditional ceramics and metal implants (Tabrizian et al., 2023).

In many organisms, the body covering is organized in concentric layers, with harder outside layers on top of softer inner tissues. This architecture allows, for instance, the armored pangolin to dissipate energy efficiently, preserving function while minimizing damage (Liu et al., 2016). One example is the dermal armor of the arapaima fish (*Arapaima gigas*), whose collagen fibrils gradually change orientation from the inner layers outward. This creates a gradient of hardness which effectively redirects puncture forces along the skin’s surface, enhancing resistance to impact without significantly restricting motion (Yang et al., 2014). This graded structural organization exemplifies systemic optimization, where multi-scale material transitions modulate how forces propagate through tissues.

This natural layering concept has also inspired the development of biomimetic alumina-based layered ceramics that mimic the damage-tolerant architecture of biological armors. Using rapid sintering, researchers have created alternating layers with tailored microstructures that deflect and stop cracks, greatly improving toughness while maintaining the stiffness and thermal stability of alumina. These ceramics show strong potential for protective coatings and high-temperature components (Prötsch et al., 2025).

Soft robotic skins also apply these biomimetic and bioinspired principles–fish scales, nacre, and dermal armor–by integrating actuation, sensing, and variable stiffness within a single adaptive layer (Shah et al., 2024). These robotic skins can transition between soft and rigid states, providing both flexibility and internal protection. Such systems mimic the mechanical behavior of living tissues and open new possibilities for adaptive robotics and wearable protective technologies.

Similar strategies are found in land animals. The protective skin of pangolins (Manidae family) and armadillos (*Armadillo* genus) consists of keratin or bony plates layered over soft tissues, forming segmented armor systems that dissipate energy and halt crack propagation (Chen et al., 2011; Wang et al., 2016). Following this natural strategy, engineers have developed biomimetic corrugated-core sandwich structures that replicate the layered and energy-dissipating architecture of natural armor (Le and Goo, 2019). For instance, they have designed a lightweight thermal protection system inspired by biological shielding mechanisms. Their system consists of a rigid outer face sheet supported by a corrugated metallic core and a softer inner layer, mimicking the multi-layered arrangement found in pangolin and armadillo armor. This design effectively distributes thermal and mechanical stresses while reducing overall weight by up to 65% compared to conventional dense panels. Such structures are now being explored for aerospace and re-entry vehicles, where maintaining strength and flexibility under extreme temperature gradients is critical.

Looking ahead, synthetic biology could enable living armor materials, in which engineered microorganisms and cells are embedded within protective composites to actively repair, secrete strengthening biopolymers, or change stiffness in response to stress. Such systems would combine graded biomimetic architecture with programmable biological functions, yielding self-healing, modular, and environmentally responsive protective surfaces far beyond current passive materials (Srubar, 2021).

#### 3.2.2 Dynamic barriers

In addition to providing passive protection, various coverings dynamically regulate their stiffness, shape, or structural behavior in response to external stimuli. For example, cephalopods can locally modulate the stiffness of their skin through muscle fiber contraction, enabling them to switch between flexibility for movement and rigidity for protection (Allen et al., 2014). Similarly, certain plant leaves or stems exhibit responsive folding when subjected to wind, touch, or mechanical strain, redistributing tension to prevent damage (Buti et al., 2025). These natural phenomena have inspired the development of reconfigurable materials capable of dynamically tuning their mechanical properties in response to environmental changes (Fei et al., 2021).

Mechanical metamaterials–engineered materials whose properties arise mainly from their designed internal architecture rather than their chemical composition–offer a powerful framework for achieving adaptability (Bertoldi et al., 2017). Researchers have designed pre-stressed mechanical metamaterials whose internal configuration can be reprogrammed to adjust stiffness, damping, and energy dissipation. By integrating tunable unit cells that respond to mechanical or thermal cues, these materials can shift between compliant and rigid states (Wang et al., 2025), much like the adaptable skins of cephalopods or the responsive tissues of plants. Such systems open new perspectives for adaptive structures in fields like soft robotics, aerospace engineering, and protective equipment.

**FIGURE 5:**
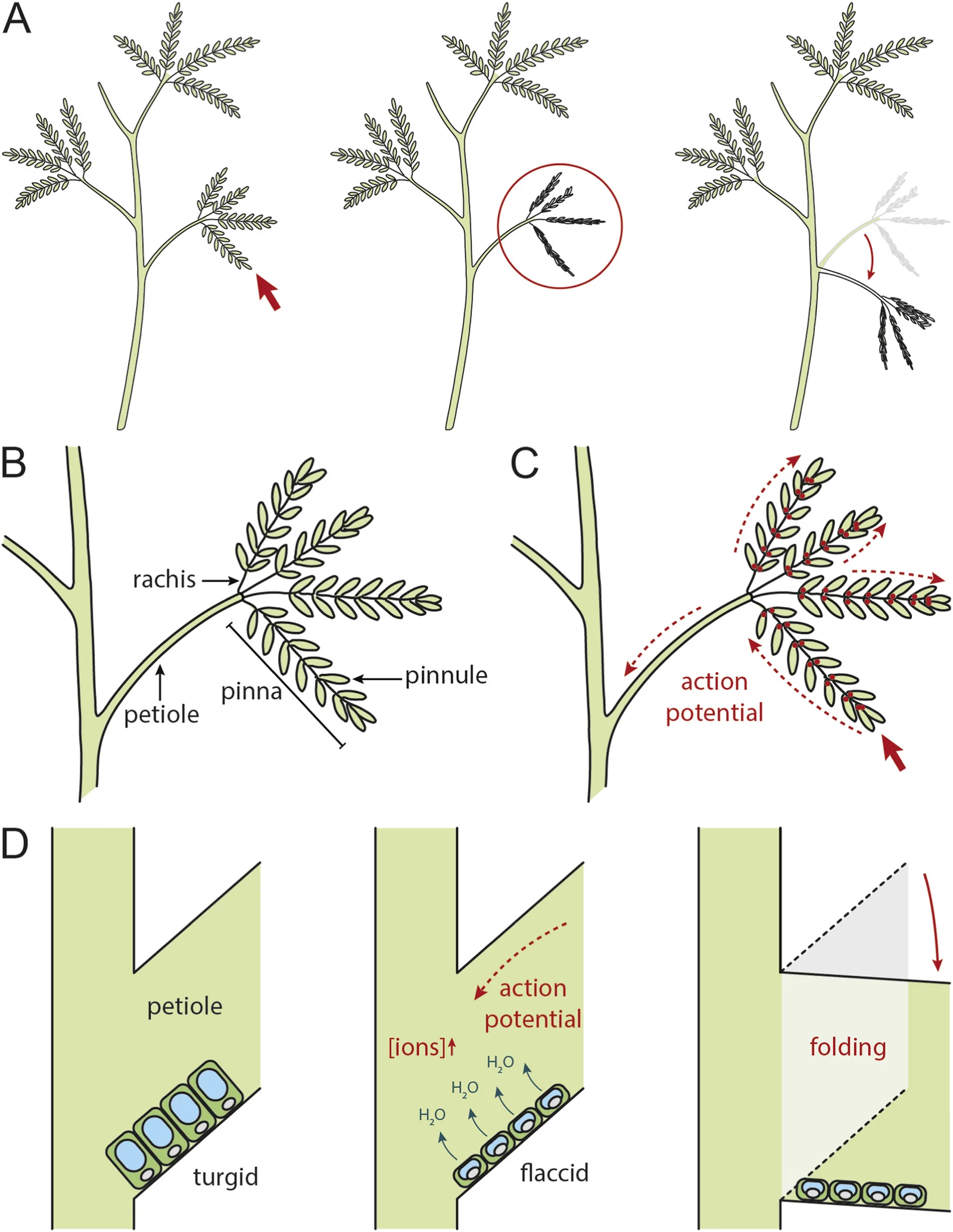
Snapping mechanism of *Mimosa pudica*. Adapted from (Kellogg, 2025) licensed under CC BY 4.0. **(A)** Leaf-folding response. A light touch (left panel) causes the pinnules (leaflets) to fold along the rachis (middle panel), whereas a stronger stimulus induces bending of the petiole (right panel). **(B)** Detailed view of the pinna and petiole. **(C)** Mechanical stimulation is detected by mechanoreceptors at the base of the pinnules. Their activation generates an electrical signal (action potential) that propagates along the rachis and petiole, analogous to signal transmission in neurons. **(D)** At the base of the petiole, the action potential triggers ion fluxes, leading to osmotic water efflux from the vacuole of motor cells. As a result, these cells lose turgor pressure, causing the petiole to bend downward.

Other designs take inspiration from the dynamic locking strategies found in various species. For example, some climbing plants like *Bauhinia* or *Mimosa pudica* have stems that can rapidly snap between two stable shapes in response to touch or bending, allowing them to fold or curl without continuous energy input (Figure 5) (Patil and Vaijapurkar, 2007). This bistable snap-through behavior arises from coordinated tissue mechanics and hydraulics, fitting within multiscale systems biology frameworks of structural and cellular interplay. Similarly, the cuticle of the locust leg joint contains folding regions that behave like origami hinges: they collapse and lock in place in order to stiffen the joint when jumping (Rajabi et al., 2022). These natural locking strategies have inspired the creation of deployable systems and morphing materials that change shape through geometry alone. Scientists developed self-deployable metamaterials made of contracting cords that can expand, fold, and stiffen without motors or heavy actuators (Yan et al., 2024). Their design allows lightweight structures to adapt to different mechanical loads with very little energy, offering new opportunities for adaptive architecture and protective devices.

The folded cuticles of insect joints also led to the design of soft robotic joints that can switch between flexible and rigid states, using origami-inspired folding patterns that twist and collapse to change stiffness (Kaufmann and Li, 2020). These systems fold and lock in place to mechanically adjust stiffness, mimicking the efficiency of natural joints. Such geometry-driven actuation enables robots to adapt their motion and stability without relying on electronics or continuous power.

When barriers rupture, quick repair is needed to avoid internal damage and further tearing of the body covering. Although repair is often linked to biological mechanisms which fall outside of the scope of this review, some coverings are also able to dynamically self-heal when damaged. For example, when scratched, the waxy cuticle of the lotus (*Nelumbo* genus) leaf wax rapidly recrystallizes by itself. This restores the leaf’s superhydrophobic surface, effectively preventing water penetration and additional damage (Wang et al., 2024). This is a systems-level phenomenon: molecular self-assembly, surface architecture, and environmental conditions interact on a multiscale level to restore functionality. Drawing from these principles, engineers have developed materials capable of restoring functionality through embedded physical responses. For instance, engineers have created biomimetic coatings based on lotus leaves that include wax-like layers. These layers can restore their water-repellent function after being scratched (Hu et al., 2024). In the same way, some structural composites are designed with self-repairing interfacial layers that absorb damage without compromising the integrity of the material. When subjected to stress, these layers yield first, dissipating energy and preventing cracks from spreading through the entire structure. After the load is removed, the interfaces reconnect at the molecular level, allowing the composite to recover its strength and stability without external intervention. This self-healing mechanism offers a durable and efficient strategy to maintain mechanical performance under repeated stress (D’Elia et al., 2016).

Synthetic biology could extend these adaptive and self-healing strategies by embedding engineered microorganisms and cells into protective materials. These living components could be programmed to sense mechanical stress, chemical damage, or temperature changes, and respond by secreting reinforcing biopolymers, crosslinkers, or lubricants. In dynamic barriers, such components could alter local rigidity on demand, emulating cephalopod and enabling protective materials that autonomously strengthen, relax, or heal themselves under real-world conditions (Srubar, 2021; Cai et al., 2025).

### 3.3 Antimicrobial barriers

Beyond purely structural defense, many organisms rely on specific antimicrobial strategies to prevent pathogen infections. These include passive mechanisms that inhibit bacterial adhesion through the composition or texture of the body covering, as well as the active release of antimicrobial agents and reactive molecules.

#### 3.3.1 Passive antimicrobial defense

In some organisms, the structure of the body covering is inhospitable to microorganisms. This constitutes a passive yet effective antimicrobial barrier. For example, the wings of cicadas (*Cicadoidea* family) and dragonflies (*Anisoptera* infraorder) feature tightly arranged, cone-shaped nanostructures called nanopillars and capable of stretching and rupturing bacterial membranes using a combination of van der Waals, electrostatic, hydrophobic, and steric forces. These nanopillar systems represent a systems biology phenomenon, as antimicrobial function emerges from the interaction of nanoscale geometry, surface chemistry, and microbial cell wall mechanics. Such structures have inspired antibacterial materials for various surfaces in hospitals, laboratories and public spaces. while reducing the use of chemical disinfectants or antibiotics (Jaggessar et al., 2017; Jin et al., 2024).

Furthermore, the body covering of various organisms possesses superhydrophobic properties, preventing dust and bacteria from settling on them (Oopath et al., 2023). Superhydrophobicity refers to the extreme water-repellent property of certain surfaces, characterized by very high contact angles between water droplets and the surface (Zhang S. et al., 2024). Roses (*Rosa* genus) are a good example of these superhydrophobic surfaces. Their petals are densely covered with micro-sized cones, each cone branching into multiple nanometer-sized folds. This pattern forms an effective superhydrophobic surface (Choo et al., 2014) which has been shown to impair biofilm formation (Cao et al., 2019). This is another systems biology example, where microscale structure, nanoscale folding, and microbial adhesion dynamics interact to produce emergent antibiofilm properties. Other examples of superhydrophobic body coverings include the gecko skin with its hair-like structures (Watson et al., 2015), as well as the shark skin, which is tiled with nanoscales (Chung et al., 2007). In all three instances, the hydrophobic surface constitutes a hostile environment for microorganisms and prevents the formation of biofilms. Similar superhydrophobic surfaces have inspired dental prosthetic materials, capable of inhibiting bacterial growth (Zhang et al., 2022).

In addition to presenting specific antimicrobial structures or superhydrophobic surfaces, some species protect themselves by embedding them into layers of protecting gel-like substances (Zhang et al., 2025). This gel-like structure enables them to protect themselves against microbial infections and environmental stress like desiccation (Olgenblum et al., 2024). Mucus is one of many examples of natural gel-like structures preventing the proliferation of bacterial biofilms thanks to their thick matrix structure and antimicrobial properties (Allen, 1983; Wagner et al., 2018). Mucus represents a systems biology barrier: its function emerges from coordinated biochemical (mucins), biophysical (viscosity, hydration), and host–microbe interactions.

Various animals secrete mucin-rich mucus at the surface of their mucosa (Benam et al., 2018). Mucins are natural glycoproteins with antimicrobial properties (Argüeso et al., 2006; Sheng and Hasnain, 2022). Therefore, in addition to preventing dryness of the mucosa, mucus secretion acts as an effective antimicrobial barrier. For example, the snail’s mucus adhesion and bioactive components such as glycolic acid and allantoin contribute to its usefulness in many areas, from wound healing to antimicrobial actions and cosmetic benefits making it a promising subject for scientific exploration (Iguchi et al., 1982; Sarkar et al., 2025). From this statement, companies such as Spartha Medical have begun developing bioinspired antimicrobial hydrogels that mimic some protective features of mucus for the healthcare industry (Tallet et al., 2021). In addition, using synthetic biology, researchers are now engineering snail mucin proteins to create artificial snail mucus with enhanced therapeutic properties (Park et al., 2023).

#### 3.3.2 Active antimicrobial strategies

In addition to presenting specific antimicrobial structures, the body of various species is covered with antimicrobial substances, which ensures chemical defense. Plant cuticles, for example, not only act as physical barriers but also contain antimicrobial compounds embedded within their structure. Recent studies have shown that cutin-derived oligomers, extracted from tomato (*Solanum lycopersicum*) peels, exhibit bactericidal effects against *Escherichia coli* and *Staphylococcus aureus* (Escórcio et al., 2022).

Some animal tissues, like bones or epithelium, can release antibacterial substances specifically at the site of infection. This active, targeted action stops potential pathogens locally, while preserving healthy cells intact (McCormick and Weinberg, 2010). Inspired by this strategy, engineers have developed surfaces that release antimicrobial agents locally and only when needed. One example is a bilayer coating made with nano-ZnO. It steadily releases zinc ions, which disrupt bacterial membranes while keeping the tissue safe. This design suits dental implants that need to prevent infection without affecting bone healing (Wang et al., 2020). Other examples include layer-by-layer coatings made from polyelectrolytes. These coatings hold antibiotics, antimicrobial peptides, or silver particles. They release their contents only when bacteria touch the surface, which avoids unnecessary exposure and keeps the surrounding tissue healthy. Such self-activating surfaces are being investigated for next-generation medical implants and wound-care materials, offering a more adaptive and safer approach to long-term infection prevention (Zhuk et al., 2014).

In addition, some natural body coverings activate their antimicrobial function in response to specific environmental conditions. For example, the surfaces of marine algae and corals generate reactive oxygen species (ROS) in response to environmental stimuli like light or oxygen exposure. These highly reactive molecules disrupt bacterial membranes and activate metabolic pathways, offering an effective antimicrobial response without requiring direct contact or the release of toxic agents (McDowell et al., 2014; Cho et al., 2022). This ROS-based activation is a systems biology mechanism linking light sensing, biochemistry, and ecological microbial regulation. One example involves a membrane made of cellulose and thermoplastic polyurethane. When illuminated, the surface generates oxidative stress that kills nearby bacteria while remaining safe for host tissue. This strategy is especially effective for medical device coatings, such as catheters or wound dressings, where infection risk is high but long-term biocompatibility is essential (Omidiyan et al., 2024).

In addition, natural surfaces have inspired antimicrobial strategies where light-activated systems produce ROS locally to target biofilms. In these contexts, antimicrobial photodynamic therapy (aPDT) uses a photosensitizer and appropriate light in the presence of oxygen to generate ROS *in situ*, which oxidize microbial cells and disrupt biofilm matrices (Li et al., 2023). Because aPDT can act on multiple microbial targets and virulence factors, it is considered a promising adjunctive approach to control biofilms on dental materials or prosthetic surfaces, while preserving biocompatibility with surrounding tissues. In parallel, photoactive hydrogels have emerged as promising materials for the treatment of chronic wounds. By embedding photosensitizers within a biocompatible polymer matrix, they can generate reactive oxygen species upon light exposure, efficiently disrupting bacterial biofilms while stimulating tissue repair, as shown in a recent *in vitro* study (Melo et al., 2025). Using this controllable photodynamic mechanism, the authors achieved efficient disinfection without harming healthy cells or promoting antimicrobial resistance. Nonetheless, as with other ROS-based therapies, careful modulation of ROS levels remains essential to avoid unintended cytotoxic effects. This promising technology will be a strong candidate for next-generation wound dressings that unite targeted sterilization with accelerated healing.

Looking ahead, synthetic biology could inspire living antimicrobial coatings, in which engineered microorganisms or cells embedded within a surface sense bacterial colonization and respond by producing antimicrobial peptides, releasing controlled bursts of ROS, or secreting protective mucin-like polymers (Srubar, 2021; Park et al., 2023).

### 3.4 Design principles emerging from defensive surfaces

Across the diverse defensive strategies reviewed in this section, common design principles emerge despite the apparent diversity of organisms and mechanisms. Species such as cephalopods (Levenson et al., 2017), chameleons (Stuart-Fox et al., 2008), chameleon prawns (Green et al., 2019), moths (Yoshida et al., 1997), and leafhoppers (Wu et al., 2025) employ stimuli-responsive and highly anisotropic optical architectures to achieve camouflage, while zebras (How and Zanker, 2014), tigers (Fennell et al., 2019), and shore crabs (Price et al., 2019) use pattern-based mechanisms whose effectiveness emerges from predator-prey interactions, illustrating emergent behavior at the ecological scale. Physical barriers such as fish scales (Chintapalli et al., 2014), nacre (Tan et al., 2019), as well as the pangolin (Wang et al., 2016) and armadillo armors (Chen et al., 2011), exemplify multiscale and graded architectures, where multiscale organization and material gradients maximize toughness while preserving flexibility. Dynamic barriers derived from cephalopod skin (Allen et al., 2014), *M. pudica* (Patil and Vaijapurkar, 2007), and the lotus leaves (Wang et al., 2024) further demonstrate the value of stimuli-responsiveness, self-healing behavior, and adaptive mechanical regulation. Antimicrobial systems found in cicadas, dragonflies (Jin et al., 2024), roses (Choo et al., 2014), geckos (Watson et al., 2015), sharks (Chung et al., 2007), snails (Sarkar et al., 2025), marine algae (Cho et al., 2022), and tomato (Escórcio et al., 2022) surfaces show how surface chemistry, micro- and nanostructure, and biological activity can be combined within modular architectures to prevent colonization, or even actively eliminate pathogens. Across these examples, biological defenses navigate trade-offs between protection, flexibility, durability, biocompatibility, and energetic cost. Such convergence suggests that effective protective systems are not defined by a single function, but rather by the integration of multiple functions within adaptive, multiscale, and multifunctional surface architectures.

## 4 Movement

Movement in biological systems is intricately linked to surface structures that have evolved to reduce drag, enable adhesion, or facilitate displacement (Guo et al., 2011). By studying the interplay between surface morphology, material properties, and movement in living organisms, researchers are developing innovative technologies, from drag-reducing coatings to soft robotic skins, that mimic and often extend the capabilities of their living counterparts. This section explores how biological surfaces contribute to movement and how these principles are being applied in biomimetic design. By integrating systems biology to model the mechanical and fluid-dynamic determinants of these surface functions, and synthetic approaches to engineer living or hybrid systems capable of tunable friction, adhesion, or drag, movement-related surface strategies can be translated into biomimetic and bioinspired interfaces (Table 3).

**TABLE 3:** Biological examples and biomimetic applications discussed in this review, related to movement.

Species	Organ/body part	Mechanism/structure	Biological phenomenon	Examples of biomimetic applications	Relevant citations for biomimetic applications
Lotus	Leaf	Micro-sized papillae; nano-sized wax crystals	Superhydrophobicity surface, drag reduction	Fluid transport systems, drag-reducing wearables, self-cleaning surfaces	Liu et al. (2024), Chen et al. (2024)
Rose	Petals	Micro-sized asperities			
Shark	Skin	Dermal denticles			
Butterfly	Wings	Ridged textures creating a stable gas-liquid interface	Waterproofing, drag reduction		
Gecko	Toes	Setae, spatulae	Dry adhesion	Synthetic adhesives	Boesel et al. (2010), Yu et al. (2011), Mengüç et al. (2012), Liu et al. (2022)
Snails	Mucus	Viscoelastic, non-Newtonian properties	Wet adhesion		
Burdock	Seeds	Hook structures at the surface of the seeds	Zoochory	Velcro and other adhesive surfaces	Saunders (2015)
Cleaver					
Enchanter’s nightshade					
Dandelion		Pappus	Anemochory	Parachutes, drug delivery systems	Yao et al. (2020), (Wang et al. (2021), Barbinta-Patrascu et al. (2024)
Maple tree	Fruits	Samara		Wind turbines, helicopter rotor blades, drones, plane components	Nave et al. (2021), Çalışkan et al. (2023)
Ash tree					

The examples listed in this table follow their order of appearance in the review.

### 4.1 Drag reduction

Various biological surfaces have evolved to allow for more efficient movement through a reduction of the friction generated by the environment (usually water or air) against their body covering (Bhushan, 2011; Liu et al., 2020). One of the most well-documented mechanisms behind this drag reduction is superhydrophobicity, a property that allows surfaces to repel water and reduce wetting that we mentioned earlier in this review. A classic example is the lotus (*Nelumbo* genus) leaf (Barthlott and Neinhuis, 1997; Pv and Kudapa, 2021; Sygouni et al., 2022; Zhang M. et al., 2024) which exhibits a dual-scale surface texture, combining micro-sized papillae with nano-sized wax crystals, that allows water droplets to bead up and roll off, carrying dirt and minimizing friction as they move. This phenomenon, known as the “lotus effect” (Barthlott and Neinhuis, 1997), illustrates systems biology principles where drag reduction emerges from interactions between nanoscale structure, microscale surface topology, and fluid dynamics, none of which alone explains the observed aerodynamic effect.

In the animal kingdom, several species have evolved similar strategies. Shark (*Selachii* division) skin, for instance, is covered with dermal denticles, which are microscopic, tooth-like scales. These denticles are arranged in riblet patterns along the body axis (Dean and Bhushan, 2010). These structures help channel water efficiently, reduce turbulence, and significantly lower drag during swimming. The surface is also naturally water-repellent, contributing to its drag-minimizing effects. Inspired by such features, engineers have developed biomimetic surfaces for anti-fouling coatings, water-repellent textiles, and efficient fluid transport systems (Liu et al., 2024).

However, superhydrophobicity is not the only mechanism by which biological surfaces reduce drag. Recent studies have turned to organisms like butterflies (*Papilionoidea* superfamily), whose wing microstructures provide compelling insights into passive friction management (Chen et al., 2024). Butterfly wings are composed of overlapping scales with ridged textures that trap a thin layer of air, creating a stable gas-liquid interface when submerged in water (Burdin et al., 2025). This air layer acts as a barrier that limits direct contact between water and the solid surface, effectively reducing drag. This drag reduction effect emerges through multilevel biological organization, from microscale scale geometry to macroscale surface aerodynamics, a hallmark of systems biology thinking.

Based on this principle, researchers have developed surfaces that replicate the multiscale morphology and spatial configuration found in natural wings. These surfaces outperformed traditional single-scale biomimetic textures in underwater drag reduction (Chen et al., 2024). Beyond drag minimization, such designs also show promise in anti-biofouling for use on boats, self-cleaning for medical surfaces, and fluidic control applications, making them attractive for a variety of industrial and environmental uses (Liu et al., 2024).

A direction yet to be explored would be the development of engineered biological coatings capable of actively regulating friction on demand by modifying their surface structure or secretions in real time. This would create a modular surface that dynamically optimizes its drag profile depending on real-time conditions, something no passive biomimetic material can do currently (Srubar, 2021).

### 4.2 Adhesion

Contrary to organisms needing less friction for better movement, various organisms maximize surface friction in order to adhere to surfaces (Gorb, 2008). Surface adhesion can be differentiated into two different categories: dry adhesion and wet adhesion.

**FIGURE 6:**
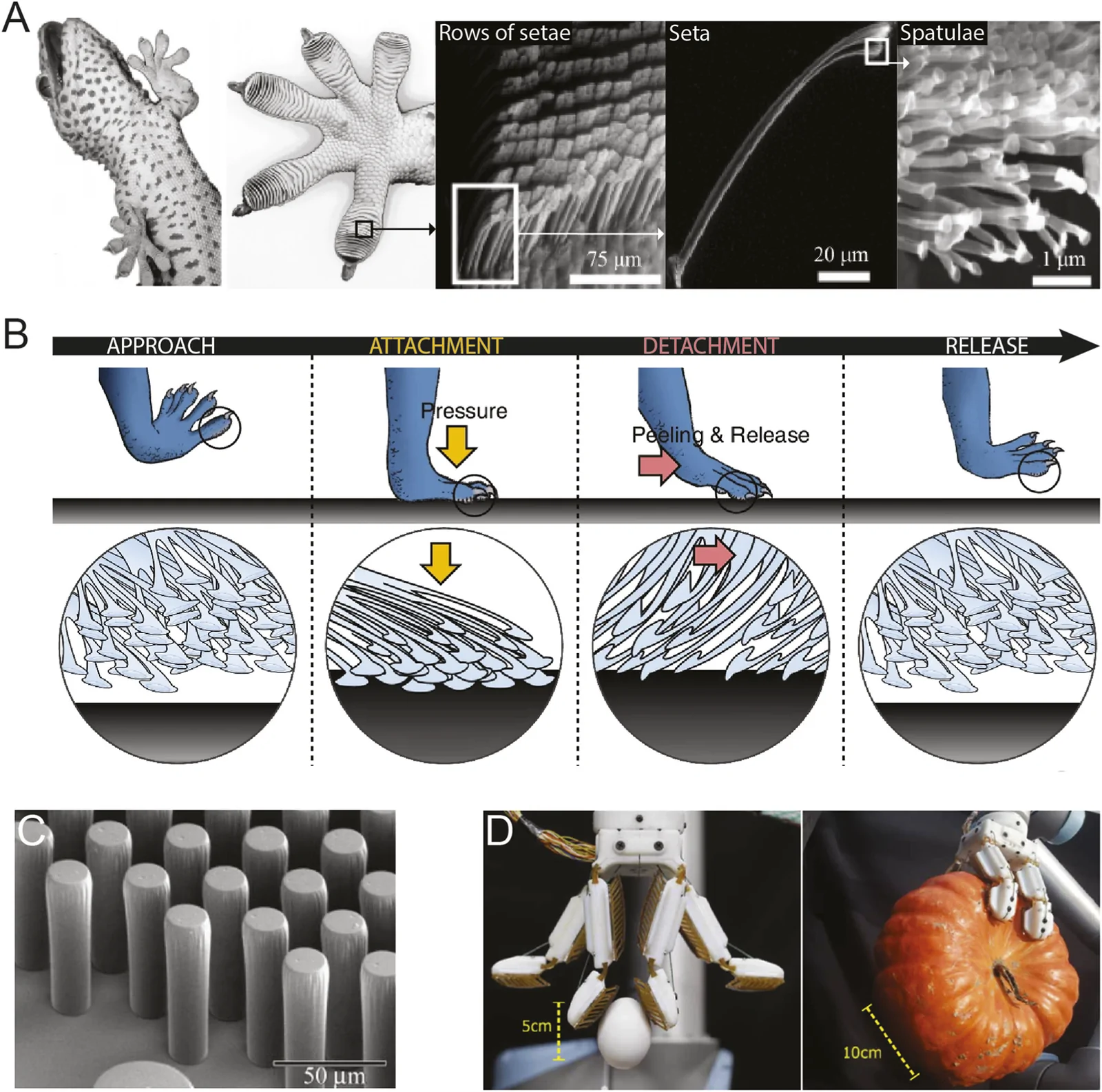
Gecko adhesion. **(A)** From left to right: ventral view of a tokay gecko (*Gekko gecko*), its foot, and three electron microscope images of a toe. Each image reveals progressively finer structural details. The toes are covered with arrays of hair-like structures called setae. Each seta branches into finer structures known as spatulae. Photo credit reproduced from (Xu et al., 2022): licensed under CC BY 4.0. **(B)** As the gecko walks, the spatulae adhere to the surface via van der Waals forces. The large number of spatulae enables strong adhesion. This adhesion is directional: the setae are angled such that, when the gecko lifts its foot, the spatulae peel off and release easily from the surface. Adapted from (Kim et al., 2025) licensed under CC BY-NC-ND 4.0. **(C)** Electron microscope image of a gecko-inspired adhesive surface. Photo credit reproduced from (Xu et al., 2022): licensed under CC BY 4.0. **(D)** Robotic arm. The inner surface of the arm is covered with gecko-inspired adhesive material, enabling a strong grip. Photo credit reproduced from (Xu et al., 2022): licensed under CC BY 4.0.

Geckos (*Gekkota* suborder) can climb walls and ceilings using a remarkable mechanism called dry adhesion, which relies on van der Waals forces rather than liquids or suction (Alibardi, 2009). Their toes are covered with millions of microscopic hairs called setae, which branch into even smaller structures called spatulae, increasing contact with surfaces at the molecular level (Figure 6A). These tiny structures allow geckos to generate enough weak intermolecular forces to support their body weight, yet they can detach easily by changing toe angles (Figure 6B). The adhesion is directional, reversible, and self-cleaning, meaning geckos can maintain stickiness even after walking through dust (Alibardi, 2009; Higham et al., 2019). This is a classic systems biology phenomenon because adhesion depends on a multiscale, graded structure, from muscular control to neural coordination and environmental interaction. Scientists are studying this system to develop synthetic adhesives for robotics, medicine, and space exploration that mimic geckos’ strong yet residue-free grip (Figures 6C,D) (Boesel et al., 2010; Yu et al., 2011; Mengüç et al., 2012; Xu et al., 2022; Kim et al., 2025).

Wet adhesion, on the other hand, relies on the presence of water or secretion between the organism and surface it wants to stick to. Snails (*Gastropoda* order), for example, use a specialized form of wet adhesion that relies on a mucus-based system to move and cling to a wide range of surfaces, including vertical and inverted ones (Fan and Gong, 2021). This mucus is a viscoelastic, non-Newtonian fluid composed primarily of water and glycoproteins, allowing it to function both as a lubricant and an adhesive for the snail, as well as helping its locomotion (Heinritz et al., 2024). The mucus forms a thin film between the snail’s muscular foot and the substrate, generating capillary and viscous forces that create strong, reversible adhesion. As the snail moves, it produces rhythmic muscular contractions called pedal waves that propel it forward while maintaining continuous contact through the mucus layer. On rough or porous surfaces, the mucus can also penetrate surface irregularities, enhancing adhesion through mechanical interlocking. This wet adhesion mechanism is highly effective in both dry and humid environments, offering low-energy, reliable movement across a variety of terrains (Zhu et al., 2024). Currently, many industrial adhesives with similar properties are made through petrochemical practices and are known to be almost impossible to degrade, making them highly unsustainable. The development of biomimetic glues (Liu et al., 2022) would allow for a more durable product as well as a product that is more easily treated when needed, without the use of intensive chemicals, while maintaining its adhesive properties.

Adhesion is not only used by animals for displacement, but also by plants as a strategic tool to spread and colonize new areas over time. Many plant species have evolved specialized seed structures that use adhesion to facilitate dispersal via zoochory, the transport of seeds by animals (Saunders, 2015). This is a systems biology example, where seed microstructures, ecological interactions with animals, and population-level dispersal dynamics form a multiscale biological system. To accomplish this, seeds are often covered with microstructures such as hooks, barbs, or sticky coatings that allow them to catch onto the body of animals passing through their habitat. These adhesive adaptations enable seeds to be carried far from the parent plant. An example of this strategy involves seeds with hook-like structures on their surfaces (Gorb and Gorb, 2002; Saunders, 2015), which anchor effectively to animal fur. Several plant species have developed this trait, including burdock (*Arctium minus*), cleavers (*Galium aparine*), and enchanter’s nightshade (*Circaea lutetiana*), all of which rely on such hooks for efficient dispersal (Gorb and Gorb, 2002). The surface morphology and mechanical behavior of these hooks were so effective that they inspired Velcro, a fastening system consisting of synthetic hooks and loops that attach and detach repeatedly (Saunders, 2015).

In addition, future designs may incorporate engineered microorganisms to create living glue systems that sense when attachment or repair is needed and respond by producing bio-adhesive proteins on demand (An et al., 2020). Previous studies demonstrated that synthetic adhesins in engineered bacteria can be quantitatively tuned to control cell-cell adhesion (Costan et al., 2024). More recently, scientists engineered bacteria that can secrete barnacle-derived adhesives in response to specific biological cues, demonstrating that synthetic gene circuits can drive living glues to repair damaged surfaces autonomously (Ge et al., 2026). Building on this foundation, such living coatings could offer self-renewing, adaptive adhesion for medical, industrial, or robotic applications.

### 4.3 Displacement

Several other methods enable plants to achieve movement through their seeds and fruits, facilitating wide dispersal and colonization of new environments. Many species have evolved structures that take advantage of local environmental forces, such as wind, gravity, and water runoff, to aid in seed displacement across great distances. From a systems biology perspective, these dispersal strategies emerge from coordinated interactions between seed morphology, fluid dynamics, and ecological selection pressures. One example is the common dandelion (*Taraxacum officinale*), whose seeds are equipped with a radial arrangement of fine bristles named pappus, that allows them to drift effortlessly on air currents (Seale et al., 2022). This pappus structure promotes the formation of stable air vortices, which effectively reduce drag and enable long-distance wind dispersal, a phenomenon called anemochory (Cummins et al., 2018). This mechanism has inspired various human technologies, such as parachutes, originally developed for military use, and continues to influence emerging designs in drug delivery systems, in the form of dandelion-like nanomicelles (Yao et al., 2020; Wang et al., 2021; Barbinta-Patrascu et al., 2024).

Similarly, many tree species use air and wind to disperse their seeds by producing samara-like fruits: this is a type of dry fruit with winged extensions that promote gliding or spinning as they fall (der Weduwen and Ruxton, 2019). Maple trees (*Acer* spp.) and ash trees (*Fraxinus* spp.) are prominent examples, with fruits shaped to maximize aerodynamic stability and reduce descent speed, allowing them to spread more widely from the parent plant (Nave et al., 2021). Their dispersal effectiveness arises from system-level tuning of mass distribution, wing geometry, and environmental airflow. These fruits have attracted attention from engineers and scientists studying passive flight and wing design, particularly for applications in wind turbines and helicopter rotor blades (Nave et al., 2021; Çalışkan et al., 2023). The morphology of samara fruits, including their wing length, curvature, and internal mass distribution, plays a critical role in determining flight behavior and dispersal range (der Weduwen and Ruxton, 2019). Continued research into these natural designs could pave the way for innovative biomimetic applications, ranging from efficient seed-planting drones to novel aerospace components and sustainable transport technologies.

### 4.4 Design principles emerging from movement-related surfaces

The examples presented in this section demonstrate that efficient movement often arises from a common set of surface-design principles operating across biological scales. The lotus leaf (Barthlott and Neinhuis, 1997) shark skin (Dean and Bhushan, 2010), and butterfly wings (Burdin et al., 2025) illustrate how multiscale and graded architectures reduce drag by combining micro- and nanoscale features that regulate fluid flow. Shark denticles (Dean and Bhushan, 2010), butterfly wing scales (Burdin et al., 2025), and gecko setae (Alibardi, 2009) further exemplify anisotropy and directionality, generating asymmetric interactions with water, air, or solid substrates that improve performance while minimizing energetic costs. The adhesive systems of geckos (Alibardi, 2009), snails (Fan and Gong, 2021), and plants such as burdock (Gorb and Gorb, 2002) highlight the importance of balancing trade-offs and Pareto fronts, particularly between adhesion and release, strength and reversibility, or persistence and mobility. Seed dispersal systems in dandelions (Seale et al., 2022), as well as maples and ash trees (Nave et al., 2021), further demonstrate how sophisticated aerodynamic performance can emerge from relatively simple geometries, reflecting principles of emergent behavior and passive optimization. Collectively, these examples illustrate how hierarchy, directionality, adaptability, and optimization repeatedly emerge as core design principles that could drive the next-generation of biomimetic surfaces and devices.

## 5 Discussion

Biological surfaces are dynamic, multifunctional interfaces that perform a wide range of critical tasks. From communication and exchange to defense and movement, these surfaces exhibit a remarkable convergence of structural ingenuity and functional efficiency. Through the lens of biomimicry, this review has explored how the study of biological surfaces can inform and inspire the design of advanced systems (Tables 1-3; Box 1).

In terms of communication and exchange, biological surfaces demonstrate how physical, chemical, and electrical signals can be transmitted with precision and purpose. These means of transmission include directional reflection in butterflies (Kinoshita et al., 2002; Berthier, 2010), hummingbirds (Doucet et al., 2006), mechanical signaling in crickets (Stritih-Peljhan and Virant-Doberlet, 2021) and shrimp (Versluis et al., 2000), and electrocommunication in electric fish (Crampton, 2019). These mechanisms have inspired innovations such as color-adaptive surfaces (Jonza et al., 1999; Minh et al., 2024) and biomimetic robotics (Ruffier et al., 2011; Worm et al., 2017; Berlinger et al., 2021). Similarly, the selective permeability of aquaporins (Verkman, 2013), the dynamic regulation of stomata (Falquetto-Gomes et al., 2024), and the targeted delivery capabilities of extracellular vesicles (Dixson et al., 2023) have led to breakthroughs in water filtration (Itoh et al., 2022), self-humidifying membranes (Park et al., 2016), and drug delivery systems (Niu et al., 2021; Feng et al., 2023).

On defensive strategies, biological surfaces offer a wide range of solutions to environmental threats: camouflage (Cuthill, 2019) and aposematism (Caro and Ruxton, 2019) transposed into adaptive textiles (Kim et al., 2021; Lychko et al., 2025) and stealth technologies (Cuthill et al., 2016; Ryan et al., 2024); the principle of physical and mechanical barriers (Wegst et al., 2015) inspiring the creation of flexible, impact-resistant materials used in robotics (Shah et al., 2024; Wang et al., 2025), aerospace (Le and Goo, 2019; Prötsch et al., 2025), and medicine (Tabrizian et al., 2023). In addition, various organisms, such as cicada, dragonflies, roses, or geckos, have body coverings that naturally prevent microorganisms from attaching or growing (Oopath et al., 2023; Jin et al., 2024). Other species, like snails, secrete gel-like substances with antimicrobial properties (Zhang et al., 2025). Last but not least, some organisms, such as plants and corals, possess active antimicrobial surfaces, which release antimicrobial substances in response to infections (McCormick and Weinberg, 2010; McDowell et al., 2014; Cho et al., 2022; Escórcio et al., 2022). These antibacterial strategies are paving the way for self-cleaning, bacteria-resistant coatings in healthcare and public infrastructure (Wang et al., 2020; Tallet et al., 2021; Zhang et al., 2022; Melo et al., 2025).

Finally, in our review, we discuss how surface morphology may enable movement. Drag reduction strategies, such as superhydrophobicity in lotus leaves (Gorb and Koch, 2014) and riblet structures on shark skin (Bhushan, 2011; Liu et al., 2020), have inspired engineered surfaces for antifouling coatings and efficient fluid transport (Liu et al., 2024). Adhesion mechanisms, like the gecko’s dry adhesion through van der Waals forces and the snail’s wet adhesion through mucus (Gorb and Koch, 2014), have inspired reversible, residue-free adhesives for robotics and medical applications (Yu et al., 2011; Mengüç et al., 2012; Fan and Gong, 2021). Plant dispersal strategies, such as hook-like seed structures for zoochory (Gorb and Gorb, 2002; Saunders, 2015) and aerodynamic adaptations like dandelion pappus and samara fruits (Nave et al., 2021; Seale et al., 2022), provide models for innovations in aerospace and drug delivery systems (Saunders, 2015; Nave et al., 2021; Çalışkan et al., 2023).

Looking ahead, the field of biomimetic surfaces stands at a promising crossroads. Advances in materials science, nanofabrication, and synthetic biology are making it increasingly feasible to replicate or even enhance natural surface functionalities. However, many biological systems operate under conditions that are difficult to reproduce in artificial settings, and the integration of multiple functions into a single material remains a formidable task. Moreover, ethical and ecological considerations must guide the translation of biological insights into technological applications.

Beyond direct inspiration from natural structures, emerging approaches in systems biology and synthetic biology are reshaping the possibilities in biomimetic surface design. Systems biology provides quantitative, multiscale models of how biological surfaces arise from genetic, biochemical, and mechanical processes, enabling the identification of key regulatory nodes and developmental trajectories that generate specific surface architectures or functions (Kitano, 2002). These principles allow bioengineers to move from simple imitation to rational designs, pinpointing the mechanisms most crucial for their needs. Synthetic biology complements this by enabling the programmable construction of living or hybrid systems, capable of producing tailored surface chemistries, dynamic morphologies, or stimuli-responsive behaviors (Endy, 2005).

Together, these disciplines represent a significant opportunity for biomimicry to not only emulate nature, but also to engineer surfaces that express biological logic in new and versatile ways. Although the biological systems reviewed here differ greatly in their taxonomy, ecology, and function, a limited set of recurring design principles emerges across communication, exchange, defense, and movement. These include multiscale and graded architectures, as illustrated by *Morpho* butterfly wings, shark skin, gecko adhesive pads, or the multiscale armor of the arapaima fish; emergent behavior, exemplified by anchovy schools, ant pheromone networks, and extracellular vesicle-mediated communication; anisotropy and directionality, which underlie optical signaling in *Morpho* butterflies and hummingbirds, drag reduction in sharks, and reversible adhesion in geckos; stimuli-responsive regulation, observed in stomata, cephalopod skin, chameleons, and *M. pudica*; and trade-offs between competing functions, such as permeability versus selectivity in aquaporins, water conservation versus gas exchange in CAM plants, or adhesion versus release in gecko and snail-inspired systems. A further recurring theme is multifunctionality, whereby a single biological surface performs several tasks simultaneously. For example, cephalopod skin integrates communication, camouflage, and environmental sensing, shark skin combines drag reduction and antifouling properties, and snail mucus acts as both a glue and antimicrobial barrier. Together, these principles constitute transferable design strategies that can inform the development of future biomimetic designs. This perspective is consistent with broader concepts of self-organization and self-assembly, which emphasize how complex functions can emerge from interactions among simpler components across multiple scales (Whitesides and Grzybowski, 2002; Hayashi, 2025). By focusing on these organizational rules rather than on individual case studies alone, inventors can move beyond imitation toward a more predictive and mechanistic framework for material innovation. To this end, we provide readers with an overview of the recurring systems and synthetic biology principles observed across the examples of the review (Box 1).

In conclusion, the study of biological surfaces offers not only a catalogue of ingenious solutions but also a methodological framework for innovation. By observing, understanding, and abstracting the strategies evolved by nature, researchers can develop materials and systems that are not only efficient and sustainable but also resilient and adaptable.
